# Insights into karyotype evolution and flower color variation from the genome assembly of wallflower (*Erysimum cheiri*)

**DOI:** 10.1093/plphys/kiag133

**Published:** 2026-03-13

**Authors:** Daozong Chen, Hui Huang, Haidong Chen, Xilin Gan, Yi Liu, Shubei Wan, Bo Zhu, Zhanjun Lu, Xianhong Ge, Qinyong Yang, Terezie Mandáková, Xinyi Guo, Chen Tan, Martin A Lysak

**Affiliations:** College of Life Sciences, Gannan Normal University, Ganzhou, China; College of Life Sciences, Gannan Normal University, Ganzhou, China; College of Life Sciences, Gannan Normal University, Ganzhou, China; College of Life Sciences, Gannan Normal University, Ganzhou, China; College of Life Sciences, Gannan Normal University, Ganzhou, China; College of Life Sciences, Gannan Normal University, Ganzhou, China; College of Life Sciences, Gannan Normal University, Ganzhou, China; College of Life Sciences, Gannan Normal University, Ganzhou, China; National Key Laboratory of Crop Genetic Improvement, National Research Center of Rapeseed Engineering and Technology, Huazhong Agricultural University, Wuhan, China; National Key Laboratory of Crop Genetic Improvement, National Research Center of Rapeseed Engineering and Technology, Huazhong Agricultural University, Wuhan, China; Central European Institute of Technology (CEITEC), Masaryk University, Brno, Czech Republic; Central European Institute of Technology (CEITEC), Masaryk University, Brno, Czech Republic; College of Life Sciences, Gannan Normal University, Ganzhou, China; Central European Institute of Technology (CEITEC), Masaryk University, Brno, Czech Republic; Department of Experimental Biology, Faculty of Science, Masaryk University, Brno, Czech Republic

## Abstract

Wallflower (*Erysimum cheiri*) belongs to the monogeneric Erysimeae tribe of the mustard family (Brassicaceae). It is widely cultivated as an ornamental garden plant and appreciated for its diverse flower colors. However, the absence of a high-quality genome has hampered research on wallflower genome evolution and the mechanisms underlying variations in flower color. Here, we assembled a nearly gap-free telomere-to-telomere genome of *E. cheiri*. The assembled genome enabled the reconstruction of genome evolution in the genus *Erysimum* (274 species), tracing the changes from the ancestral *n =* 8 genome (in *E. cheiranthoides*) to the derived genomes with 7 (in *E. nevadense*) and 6 (in *E. cheiri*) chromosome pairs. While the reduction from *n* = 8 to *n =* 7 was mediated by a nested chromosome fusion accompanied by inversions, the further decrease to *n =* 6 in *E. cheiri* resulted from an end-to-end translocation involving the other 2 nonhomologous chromosomes. Compared with other Brassicaceae species, *E. cheiri* showed a notable expansion of gene families related to secondary metabolite biosynthesis. Its flower color variation is primarily determined by the biosynthesis and accumulation of carotenoids and flavonoids. We mapped the metabolic pathways for carotenoids and flavonoids, identifying the hub genes regulating their biosynthesis. This research lays an important foundation for understanding the chromosomal and genome evolution of the Erysimeae tribe and paves the way for future investigations into genetic studies and breeding applications of *E. cheiri*.

## Teaser

A nearly complete genome of *Erysimum cheiri* offers insights into metabolic pathways underlying extensive flower color variation and sheds light on its genome evolution.

## Introduction

The Brassicaceae (Cruciferae), commonly known as the mustard family, is a medium-sized, globally distributed plant family with about 4,158 recognized species in 357 genera and 58 tribes (German et al. 2023; Al-Shehbaz 2025). The family includes species that are utilized for diverse purposes, including vegetables, oilseeds, and medicinal applications. Examples include cabbage (*Brassica rapa* and *B. oleracea*), oilseed rape (*B. napus*), camelina (*Camelina sativa*), and woad (*Isatis indigotica*). Several cruciferous species are cultivated worldwide as ornamental plants, including *Matthiola incana* (stock), *Hesperis matronalis* (dame's rocket), *Lobularia maritima* (sweet alyssum), *Aubrieta* spp. (aubrietas), *Orychophragmus violaceus* (Chinese violet cress), and wallflowers (mostly cultivars derived from *Erysimum cheiri*).

The genus *Erysimum* comprises at least 270 species with a wide range of base chromosome numbers from *x* = 6 to *x* = 17 (Warwick and Al-Shehbaz 2006; Al-Shehbaz 2025). The wallflower (*E. cheiri*) is an important ornamental plant, providing a variety of colors and pleasant fragrances, and shows considerable drought and cold tolerance (Fakhrzad et al. 2024). This species is commonly used in landscaping, as a potted flower and in rock gardens. Recent studies have shown that the species contains medicinal metabolites, such as cardenolides, which possess anti-inflammatory and anti-tumor properties. These compounds can be used to treat skin and heart diseases (Mosleh et al. 2019, 2021a; Fakhrzad et al. 2024). Previous research has focused on assembling the genomes of *E. cheiri* (Kiefer et al. 2019), *E. cheiranthoides* (Züst et al. 2020; Zhai et al. 2024), and *E. nevadense* (Zhai et al. 2024). The objectives of these studies included gene mining for important traits such as cardenolides, analyzing the reciprocal conversion between annual and polycarpic perennial flowering behaviors, and conducting phylogenetic and evolutionary studies within the Brassicaceae family. *E. cheiri* is a key ornamental species in the Brassicaceae family, known for its diverse range of flower colors and notable ornamental and economic value (Fakhrzad et al. 2024). However, as the genome of *E. cheiri* has not yet been annotated and assembled at the chromosome level, it is challenging to conduct in-depth studies of important traits such as flower color variation, as well as the regulatory mechanisms involved in the biosynthesis of cardenolides, flavonols, and anthocyanins. Furthermore, the absence of a genome sequence impedes efforts to reconstruct the origin, structure, and evolution of the genome.

*Erysimum*, one of the largest crucifer genera (Moazzeni et al. 2014), forms a monophyletic and monogeneric clade—the tribe Erysimeae (Al-Shehbaz 2012). Phylogenetic analysis of nuclear and plastome data indicates that Erysimeae are most closely related to the tribe Malcolmieae (Hendriks et al. 2023). Both tribes, along with Arabidopsideae and several other tribes, belong to the supertribe Camelinodae (German et al. 2023), also referred to as Lineage I or Clade A. Recently, Jiang et al. (2025) identified 8 chromosomes representing the common ancestral karyotype (ACBK) of 2 supertribes within the Brassicaceae: Camelinodae and Brassicodae (Lineage II). In Camelinodae, ACBK first evolved into the AKI genome (*n* = 8), which remained conserved in the Cardamineae, while ACBK transitioned into tAKI (*n* = 8) via reciprocal translocation prior to the divergence of most Camelinodae tribes. The Erysimeae tribe is no exception; the 8 chromosomes of *E. cheiranthoides* (Züst et al. 2020; Zhai et al. 2024) show the same structural arrangement as the 8 ancestral chromosomes of the tAKI genome (Jiang et al. 2025).

In this study, we constructed a chromosome-level, near telomere-to-telomere (T2T) genome of *E. cheiri* using PacBio HiFi, Hi-C, and Illumina HiSeq sequence data. We performed comprehensive gene annotation and comparative genomic analysis. We then reconstructed the karyotype evolution in *Erysimum* marked by a reduction of chromosome number, transforming the 8 ancestral chromosomes into evolutionary younger complements with 7 and 6 chromosomes. Furthermore, we integrated transcriptomic and metabolic profiles of petals from plants of 5 different flower colors to identify key genes and transcription factors associated with the synthesis of carotenoids and flavonoids. The high-quality genome assembly provides insights into the mechanisms of descending dysploidy in angiosperms and the evolution of flower color in crucifer species. The genomic data will be a valuable resource for identifying genes associated with important traits, facilitating the genetic improvement and breeding of *E. cheiri* cultivars.

## Results

### Near-complete T2T genome assembly of *E. cheiri*

**Figure 1:**
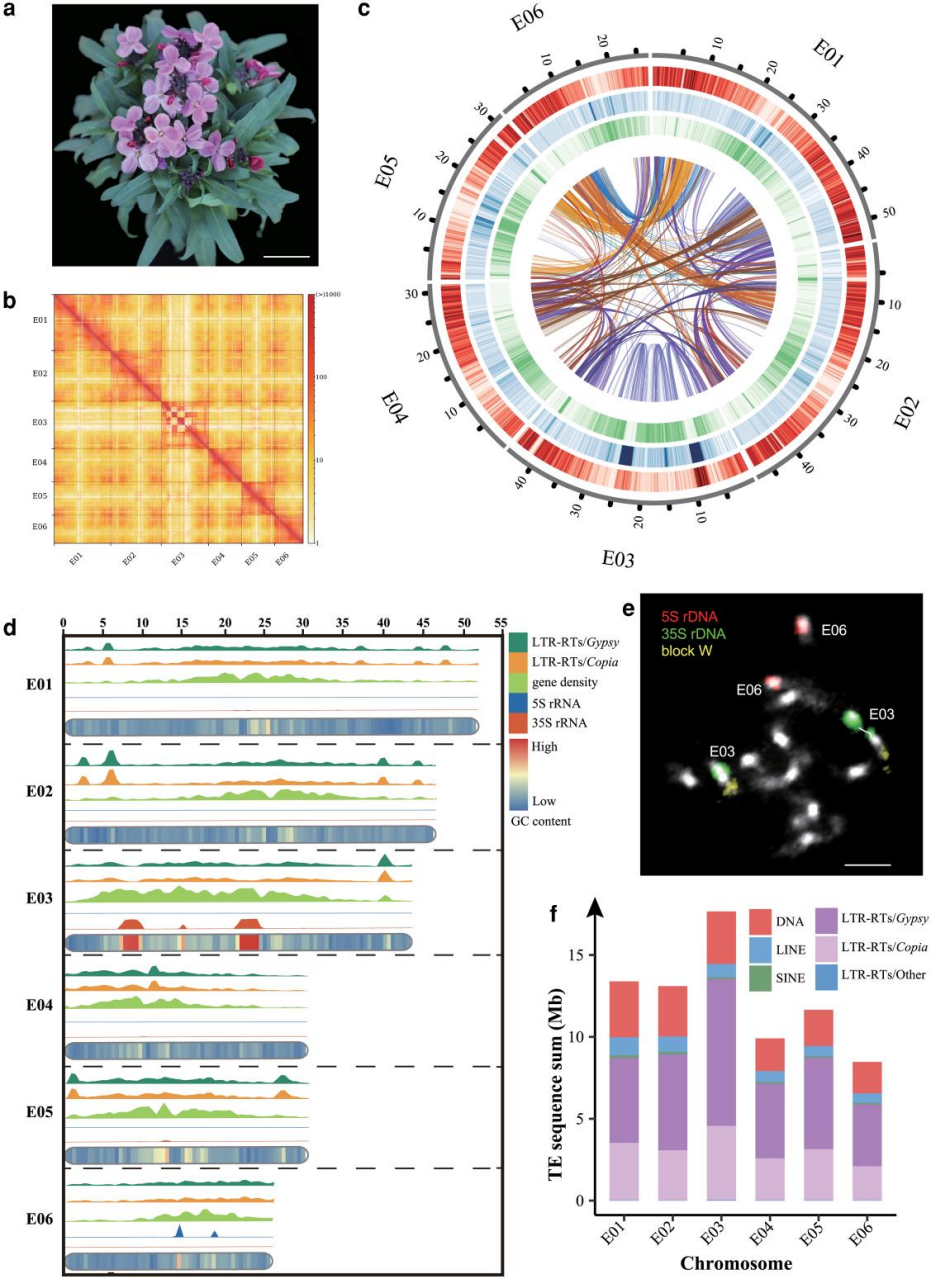
A chromosome-level genome assembly, chromosome sequence profiles, and the abundances of transposable elements in *E. cheiri*. **a)**  *E. cheiri* plant with a close-up of the purple flowers. Image was digitally extracted. Scale, 2 cm. **b)** Hi-C interaction heatmap for all 6 chromosomes. **c)** Circos plot of the *E. cheiri* genome assembly. From the outer to the inner circle are chromosome size (Mb), gene density, GC density, repeat density, and inter-chromosomal collinearity (window size 0.5 Mb). **d)** Distribution of *Gypsy*- and *Copia*-type LTR-RTs, 5S and 35S rRNA genes, gene density, and GC content along the 6 chromosomes (0.5 Mb sliding windows). The 2 peaks of 35S rDNA arrays on pseudochromosome E03 indicate the limitation of the Hifiasm assembler in assembling tandemly repeated and homogeneous sequences. **e)** Chromosomal localization of 5S and 35S rDNA on mitotic prometaphase chromosomes. Arabidopsis BAC clones from genomic block W were used to identify chromosome E03. Scale, 10 μm. **f)** The abundances of different types of transposable elements across the 6 chromosomes. DNA, DNA transposons, LINE, LINE retrotransposons, SINE, SINE retrotransposons.

To generate a high-quality genome assembly of *E. cheiri*, we used 2-mo-old young leaves of the purple-flowered plant (Fig. 1a). Using the Illumina HiSeq sequencing platform, we obtained 37.05 Gb (∼185× genome coverage) of Illumina HiSeq data to estimate genome size and heterozygosity. The *k-mer* frequency distribution (*k* = 21) indicated that the genome size of *E. cheiri* is 198.14 Mb, with a heterozygosity of 0.78% (Fig. S1). Subsequently, we obtained 47.43 Gb PacBio HiFi data (∼237×) and 94.97 Gb Hi-C data (∼475×). Additionally, we used different tissues of *E. cheiri* at different developmental stages (as described in Material and methods) to obtain 11.94 Gb of Illumina HiSeq RNA data, and 11.79 Gb of Oxford Nanopore RNA data (Table S1).

First, the draft genome was assembled using PacBio HiFi data, resulting in an assembly of 189 contigs with an N50 of 31.38 Mb, subsequently refined using Illumina HiSeq data. The Hi-C data (Fig. 1b) were then integrated for scaffolding, resulting in a 6-chromosome assembly of 244.33 Mb, with a scaffold N50 of 44.69 Mb and a GC content of 38.76% (Table 1). However, 6 gaps remained at the (peri)centromere of chromosome E03, which could not be resolved due to long arrays of repeat sequences (Fig. 1b; Table 1, Table S2). A circos plot of the T2T genome assembly was constructed to visualize chromosome size, gene density, GC density, repeat density, and inter-chromosomal collinearity (Fig. 1c). Our additional analysis revealed that the 6 gaps in the pseudochromosome E03 are primarily located in the pericentromere region enriched with 35S rDNA (Table S2, Fig. 1d), and the rDNA locus was also confirmed by fluorescence in situ hybridization (FISH, Fig. 1e). This large 35S rDNA locus complicated the process of linking contigs together to create a continuous pseudochromosome during de novo assembly. A similarly large 35S rDNA locus was identified by FISH on chromosome E03 in another accession of *E. cheiri* and 2 other *Erysimum* species (Fig. S2).

**Table 1:** Statistics for the assembly and annotation of the *E. cheiri* genome.

Assembly feature	
Estimated genome size (Mb)	198.14
No. of contigs	189
N50 of contig (Mb)	31.38 (longest: 53.35)
Genome assembly (Mb)	244.33
GC content (%)	38.76
No. of chromosomes	6
N50 of scaffold (Mb)	44.69
No. of gaps	6 (chromosome E03)
No. of telomeres	12
No. of predicted centromeres	6
Proportion of repetitive regions in the assembly (%)	43.26
Gene number	38,788
Average DS length (bp)	1,177
Average gene length (bp)	2,329
Average number exon per gene	4.7
Functional annotation (%)	97.03
BUSCO (% of complete)	99.50
LTR Assembly Index (LAI)	33.5

The lengths of the 6 chromosomes ranged from 26.79 Mb (E06) to 53.35 Mb (E01), with the fusion chromosomes E01 and E02 being the longest in the complement (Table S3). The 6 centromeres ranged in length from 0.87 Mb (E06) to 1.58 Mb (E05) (Fig. S3; Table S4), showing no clear correlation with chromosome length. The telomeric repeat sequence successfully identified all 12 telomeric regions across the 6 chromosomes (Fig. S3; Table S3).

We used both the de novo and RepBase prediction methods to annotate the repetitive sequences. The identified repetitive sequences accounted for 43.26% (105.70 Mb) of the *E. cheiri* genome. The predominant type of repetitive sequences were long terminal repeat retrotransposons (LTR-RTs), representing 24.07% of the genome (Table S5). *Gypsy*-type LTR-RTs are the most abundant repeats across the 6 chromosomes of *E. cheiri*, followed by the *Copia*-type LTR-RTs (Table S5, Fig. 1f). While *Gypsy*-type LTR-RTs are predominantly located in the pericentromeric regions, *Copia*-type elements are less common in the pericentromeres and tend to be more abundant in the gene-poor subtelomeric regions (Figs. 1d and f).

By integrating de novo predictions, homology predictions (with *Arabidopsis thaliana*, *Brassica juncea*, *B. napus*, *B. rapa*, and *Raphanus sativus*), and transcriptome predictions, we predicted 38,788 protein-coding genes (Table S6). To evaluate the consistency of the genome, the reads obtained from second-generation sequencing were aligned to the assembled genome. The results indicated an overall alignment rate of 99.50%, encompassing 99.80% of the annotated genes (Table S7). The BUSCO analysis revealed that 99.50% of genes were completely assembled, thereby indicating an overall robust integrity of the genome assembly (Table S7). The *k-mer* statistical analysis yielded a QV value of 45.47 for the genome, with individual chromosome QV values ranging from 47.61 to 54.73 (Table S8), suggesting a high accuracy of the genome assembly. Additionally, the LTR assembly index (LAI) for the *E. cheiri* genome was 33.50 (Table 1). Collectively, these results indicate that the assembled *E. cheiri* genome is of high quality, reliable, and accurate.

### Gene family, phylogenetic relationship, and genome collinearity analysis

**Figure 2:**
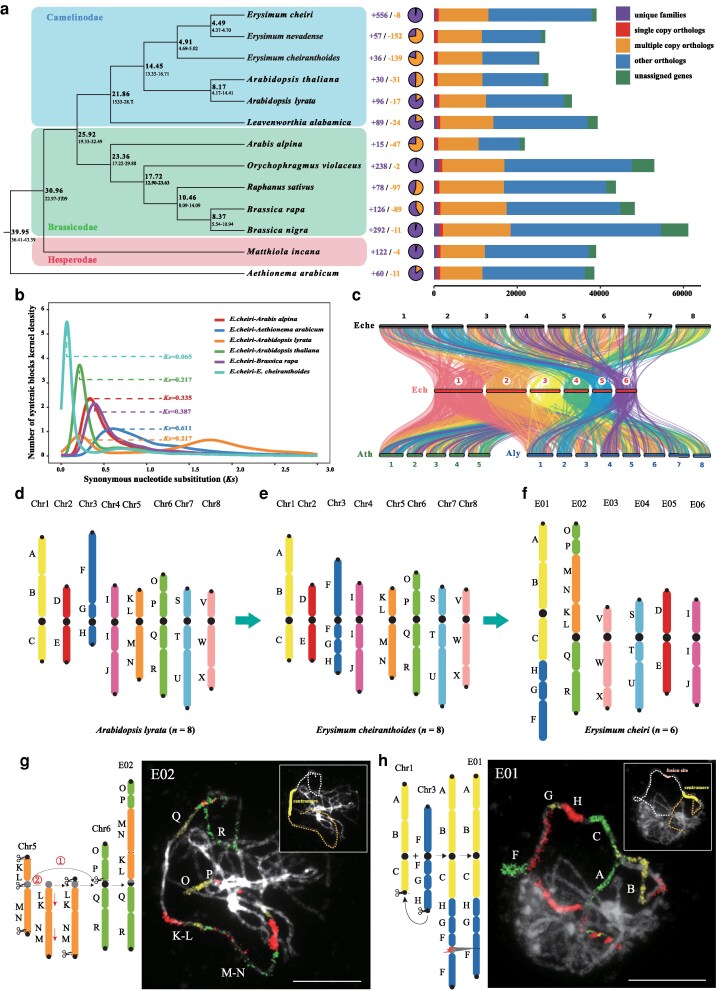
Evolutionary and comparative analysis of the *E. cheiri* genome. **a)** The phylogenetic tree and estimation of divergence time for *E. cheiri* and the other 12 crucifer species. The middle panel displays the expansion/contraction of gene families; the right panel displays the distribution of gene classes. The numbers at the nodes of the tree represent divergence time with units millon years ago (Mya). **b)** The distribution of *Ks* values among colinear genes between *E. cheiri* and 6 crucifer species. **c)** The syntenic diagram between *E. cheiri* (Ech) and 3 crucifer species, *E. cheiranthoides* (Eche), *A. thaliana* (Ath), and *A. lyrata* (Aly). **d–f)** Ideograms of chromosome-level genome assemblies of *A. lyrata* (*n* = 8), *E. cheiranthoides* (*n* = 8) and *E. cheiri* (*n* = 6) showing the 22 conserved genomic blocks (A to X) of crucifer genomes (Lysak et al. 2016). The arrows indicate the likely direction of karyotype evolution, with the *A. lyrata* genome serving as a proxy for the ancestral tAKI genome (*n* = 8). **g)** The inferred origin of chromosome E02 in *E. cheiri* from the ancestral chromosomes chr5 and chr6 via a nested chromosome fusion, and comparative chromosome painting of fusion chromosome E02 in pachytene (meiosis I). Two alternative origins of E02 are proposed. The first ① suggests that the fusion chromosome originated as a result of 4 double-strand breaks (DSBs) on the 2 ancestral chromosomes; the second ② assumes that chromosome chr5 underwent one pericentric and one paracentric inversion, resulting in a telocentric chromosome, followed by NCF with chromosome chr6. **h)** The inferred origin of chromosome E01 in *E. cheiri* via an end-to-end translocation between the ancestral chromosomes chr1 and chr3, and comparative chromosome painting of fusion chromosome E01 in pachytene. Three different fluorochromes were used to distinguish the genomic blocks: Cy 3 (yellow), Alexa 488 (green), and Texas Red (red). Chromosomes were counterstained with DAPI; condensed heterochromatic regions are visible as bright/white arrays. Scales, 10 μm.

Together with *E. cheiri*, 12 genomes representing supertribes Arabodae, Brassicodae, Camelinodae, and Hesperodae (*E. cheiranthoides*, *E. nevadense*, *A. thaliana*, *A. lyrata*, *Leavenworthia alabamica*, *Arabis alpina*, *Orychophragmus violaceus*, *R. sativus*, *B. rapa*, *B. nigra*, *Matthiola incana*) and the Aethionemoideae (*Aethionema arabicum*), were selected for homologous gene identification, gene family clustering analysis, and enrichment of single-copy and multi-copy genes (Fig. 2a; Table S9). A total of 63,075 orthologous gene families, comprising 489,573 genes, were identified across all 13 species; 7,524 gene families, comprising 171,981 genes, were common to all species. The genome of *E. cheiri* harbored 298 unique gene families, which included 1,720 unique paralogs (Tables S10 to 11). GO enrichment analysis revealed that these unique genes were predominantly associated with various biological processes, such as RNA-directed 5 to 3 RNA polymerase activity, cytoplasmic functions and responses to toxic substances. These genes were notably enriched in pathways related to the defense response to fungi, responses to toxic substances, the killing of cells from other organisms, and responses to cold (Fig. S4; Table S12). Further analysis using the KEGG indicated that these unique genes were involved in exopolysaccharide biosynthesis, lipoic acid metabolism and the citrate cycle pathways (Fig. S5; Table S13). Importantly, these genes were also enriched in pathways related to the defense response to fungi and the biosynthesis of unsaturated fatty acids (Fig. S5; Tables S12 to 13), aligning with previous studies indicating that *E. cheiri* possesses anti-inflammatory properties and a high unsaturated fatty acid content in its seeds (Mosleh et al. 2021a, 2021b).

Gene family expansion and contraction analysis revealed that 556 gene families in *E. cheiri* underwent significant expansion, whereas only 8 gene families experienced contraction (Table S14). The expanded gene families were apparently enriched in GO and KEGG pathways. GO analysis indicated that the expanded genes in *E. cheiri* were involved in various biological processes, including RNA-directed DNA polymerase activity, respiratory chain functions, and DNA integration. Notably, pathways associated with plant defense, such as the killing of cells from other organisms and the defense response to fungus, were also enriched (Fig. S6; Table S15). KEGG enrichment analysis further demonstrated significant enrichment in pathways related to the biosynthesis of various plant secondary metabolites, as well as tropane, piperidine, and pyridine alkaloid biosynthesis (Fig. S7; Table S16). These expanded gene families could result from independent adaptive evolution and long-term selection by humans for ornamental and medicinal plants. Subsequently, we used 819 single-copy genes to estimate the divergence times among 13 species and constructed a phylogenetic tree (Fig. 2a; Table S10). The calculations revealed that the common ancestor of Camelinodae, Brassicodae, and Hesperodae diverged from *Ae. arabicum* (Aethionemoideae) approximately 39.95 millions years ago (Mya). Camelinodae diverged from Brassicodae ∼21.86 Mya, *Erysimum* diverged from *Arabidopsis* ∼14.45 Mya, and *E. cheiri* ultimately diverged from *E. nevadense* ∼4.49 Mya.

Additionally, we selected 6 representative Brassicaceae species to analyze their relationship with *E. cheiri* in terms of syntenic blocks, kernel density and the synonymous substitution rate (*Ks*) of orthologous genes. The results showed that when the density value between *E. cheiri* and *E. cheiranthoides*, *A. lyrata*, *A. thaliana*, *Ar. alpina, B. rapa*, and *Ae. arabicum* was at its maximum, the peak values of *Ks* for orthologous genes were 0.065, 0.217, 0.217, 0.335, 0.387, and 0.611, respectively. This suggests that a closer species relationship correlates with a lower synonymous substitution rate (Fig. 2b). Furthermore, to evaluate the collinearity between the *E. cheiri* genome and its closely related species, we compared the gene sequences of *E. cheiri* with those of 3 closely related species: *A. thaliana*, *A. lyrata*, and *E. cheiranthoides*. The results demonstrated that *E. cheiri* exhibited strong genomic collinearity with *E. cheiranthoides* (Fig. 2c).

### The origin and evolution of the *E. cheiri* genome

As in many Camelinodae tribes, the Erysimeae genomes (Fig. S8) are thought to originate from the ancestral tAKI genome (*n* = 8), structurally similar to *Arabidopsis n =* 8 genomes or *Capsella rubella* (Jiang et al. 2025). Therefore, we first compared the genomes of *A. lyrata* (*n* = 8) and *E. cheiranthoides* (*n* = 8; Züst et al. 2020; Zhai et al. 2024). This comparison confirmed the tAKI-like structure of the *E. cheiranthoides* genome with the exception of chromosome 3 (Fig. 2d and e). Whereas the ancestral position of centromere 3 in tAKI (chromosome Al03 in *A. lyrata*) is between blocks G and H, the centromere is located within genomic block F in *E. cheiranthoides* (Figs. S9 to 10). The preserved collinearity between chromosome tAKI_3 (Al03) and chromosome 3 in *E. cheiranthoides* points to centromere repositioning in the ancestral *Erysimum* (*n* = 8) genome (Fig. S10). The centromere has shifted from its original location between blocks G and H to block F (Fig. 2e), a repositioning of ∼3.6 Mb.

The available assembly of the *E. cheiranthoides* genome allowed us to reconstruct the origin of the 6-chromosome genome of *E. cheiri* (Fig. S11). To this end, we also used the contig-level genome assembly of *E. nevadense* with 7 chromosome pairs (Zhai et al. 2024). While 4 chromosomes (E03, E04, E05, and E06) in *E. cheiri* are collinear with their homoeologs in *E. cheiranthoides*, the 2 longest chromosomes—E01 and E02—originated by descending dysploidy from *n* = 8 (*n* = 7). The 6 assembled pseudochromosomes were orthogonally validated by comparative chromosome painting using *A. thaliana* BAC contigs, confirming the correct assembly of the 2 fusion chromosomes by the congruent chromosomal position of homoeologous genomic blocks (Figs. 2f to h).

Both *E. cheiri* and *E. nevadense* share a chromosome fusion event superficially resembling a nested chromosome fusion (NCF) combining ancestral chromosomes 5 and 6 into chromosome E02 (Fig. 2g). The origin of fusion chromosome E02 was not a simple NCF because 2 arms of the inserted chromosome (chr5) do not have the ancestral telomere-to-centromere orientation, and its centromere is not the active one. The NCF event can be reconstructed by assuming that 4 double-strand breaks (DSBs) occurred on the inserted (chr5) and recipient (chr6) chromosomes. Three DSBs at the telomeres and pericentromere of the inserted chromosome (chr5) and 1 DSB at the pericentromere of the recipient chromosome (chr6) facilitate the rejoining of the broken chromosomes with the altered orientation of the arms of the inserted chromosome. Alternatively, the NCF was preceded by pericentric and paracentric inversions that inverted both arms of chromosome 5, rendering this chromosome telocentric. In both scenarios, ectopic recombination may have resulted in the formation of a hybrid 5/6 centromere on the fusion chromosome E02 (Fig. 2g and Figs. S12 to 15). The origin of E02 aligns with the asymmetric + donor arm repositioning model of NCF (Luo et al. 2009; Fig. 3b in Schubert and Lysak 2011).

**Figure 3:**
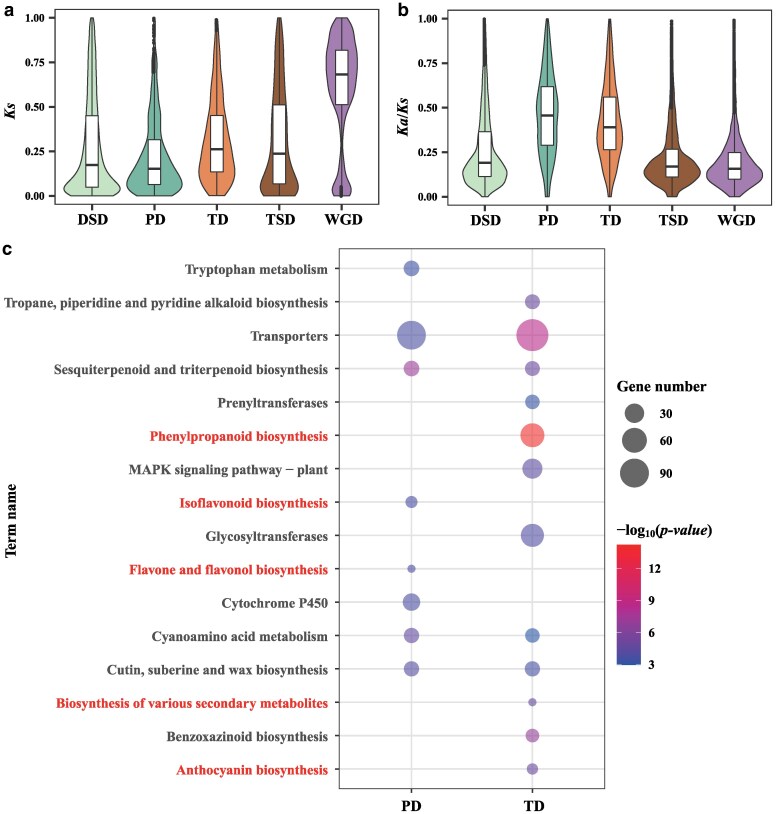
Analysis of duplicated genes. **a)** The distribution frequencies of synonymous nucleotide substitutions (*Ks*) for 5 types of gene duplication. **b)**  *Ka*/*Ks* values of gene pairs derived from 5 types of gene duplication. In the violin plots, the center black lines represent the medians, the box limits correspond to the 25th and 75th percentiles, the whiskers extend to 1.5 times the interquartile ranges, the shapes indicate the data distributions. **c)** KEGG enrichment analyzes of PD and TD-type genes. The details are listed in Table S18. Pathways related to the metabolism of carotenoids, flavonoids, and anthocyanins are highlighted in red. DSD, dispersed duplicated genes, PD, proximal duplicated genes, TD, tandem duplicated genes, TSD, transposed duplicated genes, WGD, whole-genome duplicates.

A further descending dysploidy was mediated by an end-to-end chromosome translocation between ancestral chromosomes 1 and 3 to form the longest chromosome E01 (Fig. 2h, Fig. S16a). The fusion chromosome retained centromere 1 as the active one, whereas the centromere 3 within block F was deleted. A detailed examination of the paleocentromeric site on the bottom arm of E01 revealed no remnants of (peri)centromeric repeats within block F (Fig. S16b).

We profiled the fusion chromosomes E01 and E02 and specifically examined the breakpoint regions for increased accumulation of repetitive elements. While we did notice a locally increased abundance of several repeat subtypes (eg, *Copia*-type LTR-RTs near the P-M junction, see Figs. S17 to 18), we did not observe colocalization of increased TE abundance with the chromosome breakpoints (Figs. S17 to 19).

### Analysis of duplicated genes

*E. cheiranthoides* was the first *Erysimum* species for which a reference genome is available at the chromosome level, with an assembled genome size of 174.5 Mb and a total of 29,947 annotated gene models (Züst et al. 2020). Zhai et al. (2024) reassembled the genome of *E. cheiranthoides* and assembled the E. nevadense genome, which are 167.44 Mb and 205.05 Mb in size, respectively.. The number of protein-coding genes for these species is 25,234 and 26,641, respectively. The genome size of *E. cheiri* was estimated to be 244.33 Mb, with 38,788 predicted genes. This is markedly higher than in *E. cheiranthoides* and *E. nevadense* in terms of both genome size and number of protein-coding genes. The evolutionary analysis showed that the chromosomal structures of the *E. cheiranthoides* and *E. cheiri* genomes are well collinear, and no whole-genome duplication events were detected. Gene duplication is the predominant mode of gene expansion. To investigate the reasons for the increase of over 8,000 genes in *E. cheiri* compared to *E. cheiranthoides* and *E. nevadense*, we conducted a comprehensive whole-genome identification and analysis of duplicate genes in *E. cheiri*. The results revealed that the identified dispersed duplicated genes (DSD), proximal duplicated genes (PD), tandem duplicated genes (TD), transposed duplicated genes (TSD), and whole-genome duplicates (WGD) amounted to 11,505 (29.66%), 3,136 (8.08%), 3,325 (8.57%), 7,252 (18.70%), and 8,646 (22.29%), respectively (Table S17).

We then performed nonsynonymous nucleotide substitution (*Ka*), synonymous nucleotide substitution (*Ks*), *Ka*/*Ks*, and KEGG analyzes for these 5 types of duplicated genes. The results of *Ka* analysis showed that TD had the highest median nonsynonymous substitution rate, indicating that nonsynonymous substitutions had a greater impact on its evolution (Fig. S20). The results of the *Ks* analysis also showed that WGD had the highest median synonymous substitution rate, indicating that synonymous substitutions had a greater impact on the evolution of TD-derived genes. (Fig. 3a). The *Ka*/*Ks* analysis showed that PD had the highest median value, followed by TD, indicating that these 2 types of genes were subject to the greatest selection pressure, but the ratios of the 5 types of duplicate genes were all less than 1, suggesting that these genes were mainly subject to purifying selection during evolution (Fig. 3b). To explore the functional enrichment of the duplicated PD- and TD-type genes, we performed KEGG analysis (Fig. 3c; Table S18). The results showed that these duplicates had the most enriched genes in the transporter pathway, PD was uniquely enriched in the flavone and flavonol biosynthesis, isoflavonoid biosynthesis, and tryptophan metabolism, while TD was uniquely enriched in metabolic pathways such as phenylpropanoid biosynthesis, biosynthesis of various secondary metabolites, and anthocyanin biosynthesis. The phenylpropanoid, flavonoid, and anthocyanin metabolic pathways are closely related to plant stress defense and plant coloration, suggesting that the PD- and TD-type genes may be the result of long-term natural selection and artificial domestication. Notably, 681 of the 3,136 PD-type genes are expanded genes, and all 3,325 TD-type genes are expanded genes (Table S19), indicating that TD-type gene expansion may be key to the divergence and adaptive evolution of *E. cheiri*.

### Carotenoids and flavonoids determine the flower color of *E. cheiri*

**Figure 4:**
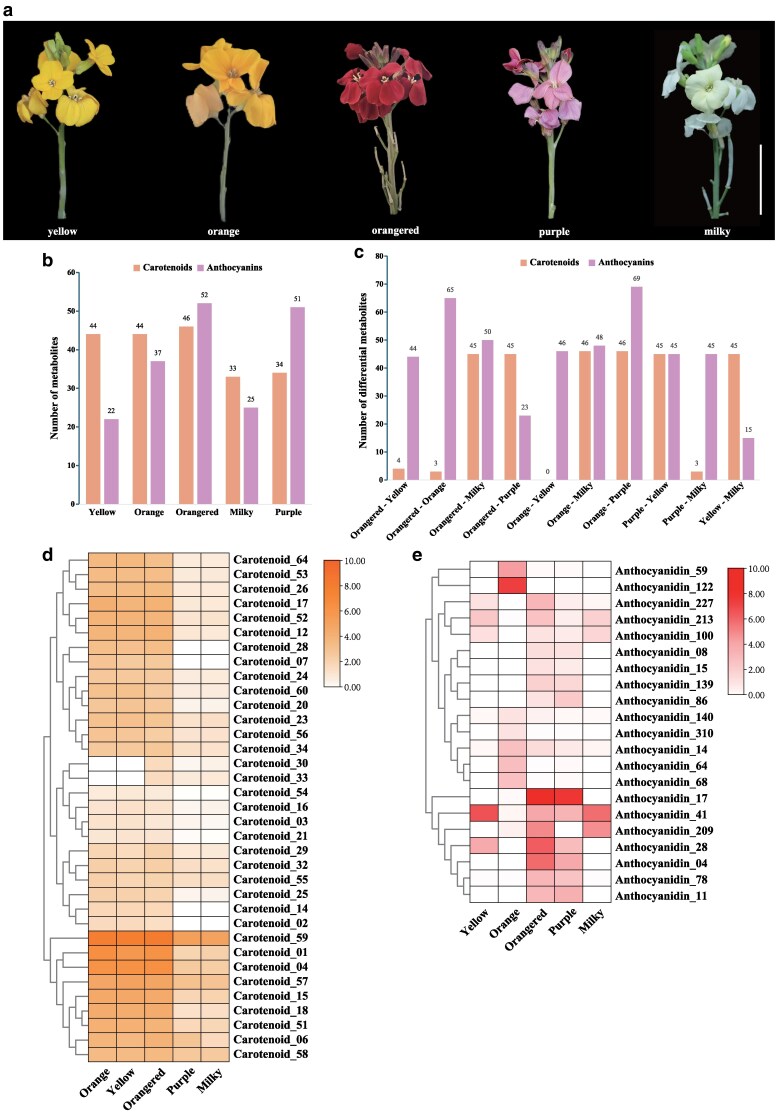
Phenotypes and metabolome analysis in *E. cheiri* plants with 5 flower colors. **a)** The phenotypes of plants with 5 different petal colors: yellow, orange, orangered, milky, and purple. Images were digitally extracted for comparison. Scale, 2 cm. **b)** The metabolites detected by targeted metabolic profiling of flavonoids and carotenoids in plants with 5 different petal colors. **c)** Differentially expressed flavonoid and carotenoid metabolites in plants with 5 different petal colors. **d)** A clustered heat map of carotenoid content (μg/g dry weight) in plants with 5 different petal colors. **e)** A clustered heat map of flavonoid content in plants with 5 different petal colors. **d)** and **e)** show contents > 1 μg/g. The complete information on metabolites is listed in Table S20.

*E. cheiri* is a popular ornamental plant widely cultivated for its lush and fragrant flowers. Many cultivars have been developed in yellow, orange, red, maroon, purple, white, and cream colors (Moazzeni et al. 2014). To investigate the changes in metabolites associated with the different flower colors, a targeted metabolic analysis of flavonoids (flavonols and anthocyanins) and carotenoids was performed in plants with 5 different petal colors: yellow, orange, orangered, milky, and purple (Fig. 4a). A total of 78 flavonoid and 68 carotenoid metabolites were identified (Table S20). Specifically, the yellow, orange, orangered, milky, and purple petals contained 44, 44, 46, 33, and 34 carotenoid metabolites and 22, 37, 52, 25, and 51 flavonoid metabolites, respectively (Fig. 4b). The 78 identified flavonoids and 68 carotenoids were clustered using the *K-means* algorithm. The flavonoids were categorized into 10 distinct clusters, while the carotenoids were divided into 9 clusters. Notably, the 10 subclasses of flavonoids showed clear differences in their trends across the 5 color groups, whereas the 9 subclasses of carotenoids exhibited relatively consistent trends across these same groups (Figs. S21 to 22). Previous studies have demonstrated that flavonoids and carotenoids accumulate in yellow petals, leading to a transition from orange to orangered, while flavonoids accumulate in white petals, resulting in a shift from purple to blue (Tanaka et al. 2008). Consequently, this study focuses on the differential metabolites (DEMs) among 3 flower color backgrounds: yellow (including orange and orangered), white (comprising milky and purple), and the comparison between yellow and milky flower color groups. The DEMs identified between yellow, orange, and orangered flowers are predominantly flavonoids, similar to those observed between milky and purple flowers, whereas the DEMs between yellow and milky flowers are primarily composed of carotenoids (Fig. 4c). The comprehensive *K-means* and DEMs analysis revealed that yellow, orange, and orange are grouped within the same color system, while milky and purple are categorized in another distinct color system (Figs. S21 to 22). This suggests that the main metabolites that determine the yellow and milky colors are carotenoids, while the colors changes of orange, orangered, and purple are mainly due to the synthesis and accumulation of flavonoid metabolites.

We screened metabolites with a content of ≥1 µg/g, generated a heat map and conducted a cluster analysis based on metabolite types. The analysis of carotenoid content revealed that the levels of lutein (carotenoid_59), β-carotene (carotenoid_01), and α-carotene (carotenoid_04) were higher in yellow, orange and orangered petals than in milky and purple petals, which could be primarily responsible for the yellow color (Fig. 4d; Table S20). The analysis of flavonoids showed that yellow petals had increased levels of quercetin-3-*O*-glucoside (anthocyanidin_41) and delphinidin-3-*O*-rhamnoside (anthocyanidin_28). In contrast, orange petals synthesized higher amounts of cyanidin-3-*O*-(6″-*O*-coumaryl)galactoside (anthocyanidin_122) and pelargonidin-3-*O*-glucoside (anthocyanidin_59), while orangered petals contained higher concentrations of cyanidin-3-*O*-sambubioside-5-*O*-glucoside (anthocyanidin_17), delphinidin-3-*O*-rhamnoside (anthocyanidin_28), cyanidin-3,5-*O*-diglucoside (anthocyanidin_04), and delphinidin-5-*O*-rhamnoside (anthocyanidin_209). Purple petals had a higher content of cyanidin-3-*O*-sambubioside-5-*O*-glucoside (anthocyanidin_17), whereas milky petals had an increased content of quercetin-3-*O*-glucoside (anthocyanidin_41) (Fig. 4e; Table S20).

### Secondary metabolites and anthocyanin biosynthetic pathway play an important role in flower color variation in *E. cheiri*

**Figure 5:**
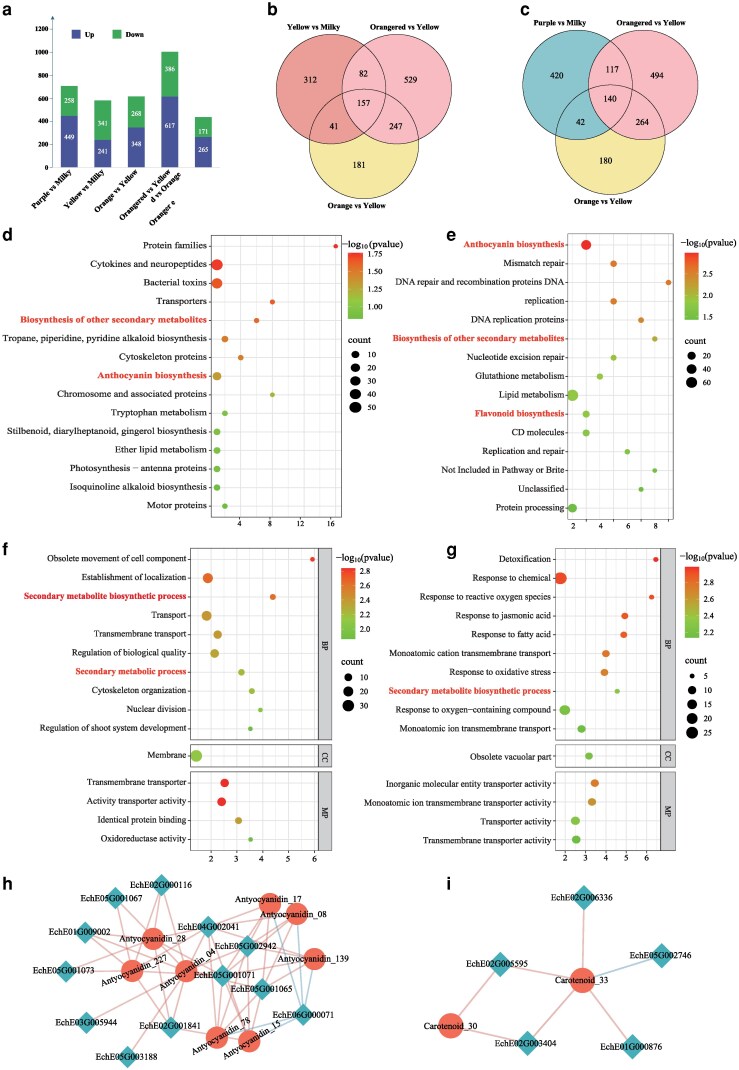
Comparative transcriptome analysis of *E. cheiri* plants with 5 flower colors. **a)** Statistics of upregulated and downregulated DEGs among plants with 5 flower colors. **b)** Venn diagrams of DEGs in yellow, milky, orange, and orangered flower petals, with carotenoids involved in the formation of these color differences. **c)** Venn diagrams of DEGs in purple, milky, yellow, orange, and orangred flower petals, with anthocyanins involved in the formation of these color differences. **d)** KEGG analysis of the top enrichment terms among the total DEGs in petals of yellow, milky, orange, and orangered flowers. **e)** KEGG analysis of the top enrichment terms among the total DEGs in petals of purple, milky, yellow, orange, and orangered flowers. The anthocyanin biosynthesis, biosynthesis of other secondary metabolites and flavonoid biosynthesis are indicated in red font. **f)** The top GO terms for total DEGs in petals of yellow, milky, orange, and orangered flowers. **g)** The top GO terms for total DEGs in petals of purple, milky, yellow, orange, and orangered flowers. The secondary metabolite biosynthetic process is emphasized in red font. The correlation network of hub genes and metabolites in the flavonoid **h)** and carotenoid **i)** biosynthetic pathways. Orange indicates a positive correlation, while blue indicates a negative correlation.

To elucidate the mechanism of flower color variation in *E. cheiri*, we conducted a comparative transcriptome analysis on the unopened flower buds of plants with 5 different petal colors. A total of 3,344 differentially expressed genes (DEGs) were identified in the 5 groups, with 707, 582, 616, 1,003, and 436 DEGs for purple-milky, yellow-milky, orange-yellow, orangered-yellow, and orangered-orange, respectively (Fig. 5a; Table S21). To investigate the differences in flower color due to the synthesis of carotenoids and flavonoids, we analyzed the common DEGs between the different groups. The results showed that there were 157 shared DEGs in the yellow-milky, orange-yellow, and orangered-yellow groups (Fig. 5b), while 140 DEGs were identified in the purple-milky, orange-yellow, and orangered-yellow groups (Fig. 5c). KEGG analysis revealed that the 157 common DEGs associated with carotenoid synthesis were enriched in biosynthesis of other secondary metabolites and anthocyanin biosynthetic pathways, whereas the 140 common DEGs related to flavonoid synthesis were enriched in anthocyanin biosynthesis, biosynthesis of other secondary metabolites, and flavonoid biosynthesis (Figs. 5d and e). Additionally, GO enrichment analysis demonstrated that these genes were highly correlated with secondary metabolic processes, with the 157 common DEGs associated with carotenoid synthesis also enriched in secondary metabolic processes (Figs. 5f and g). These findings suggest that carotenoids and flavonoids play a dominant role in determining petal color variation in *E. cheiri*.

Furthermore, we conducted RNA sequencing (RNA-seq) analysis on petals exhibiting 5 distinct colors to correlate gene expression levels with flavonoid and carotenoid content. The results indicated that flavonoids were markedly enriched in the light green and green modules (Fig. S23), whereas carotenoids were predominantly enriched in the green-yellow module (Fig. S24). Additionally, we constructed a correlation network linking hub genes and metabolites within the flavonoid and carotenoid biosynthetic pathways. In the flavonoid metabolic pathway, 12 genes were strongly associated with 7 flavonoid metabolites (Fig. 5h). Our further analysis revealed clear (differences) differences in the contents of these 7 flavonoid metabolites across the 5 petal colors (Table S20). The RNA-seq analysis confirmed that these 12 genes exhibited significant differential expression among the 5 petal colors (Table S21). In the carotenoid metabolic pathway, 5 genes were found to have strong associations with Carotenoid_33 (violaxanthin-myristate-caprate) and Carotenoid_30 (violaxanthin palmitate) (Fig. 5i). Notably, Carotenoid_33 and Carotenoid_30 were detected exclusively in orange, milky, and purple petals, suggesting that they may play a crucial role in flower color variation (Table S20). The RNA-seq analysis further revealed significant differential expression of these 5 genes across the 5 petal colors (Table S21). These findings underscore the integrity and functional importance of both the carotenoid and flavonoid biosynthetic pathways in *E. cheiri*, contributing to its remarkable variation in flower color.

### Expression patterns of carotenoid and flavonoid metabolic pathway genes in the petals of *E. cheiri*

**Figure 6:**
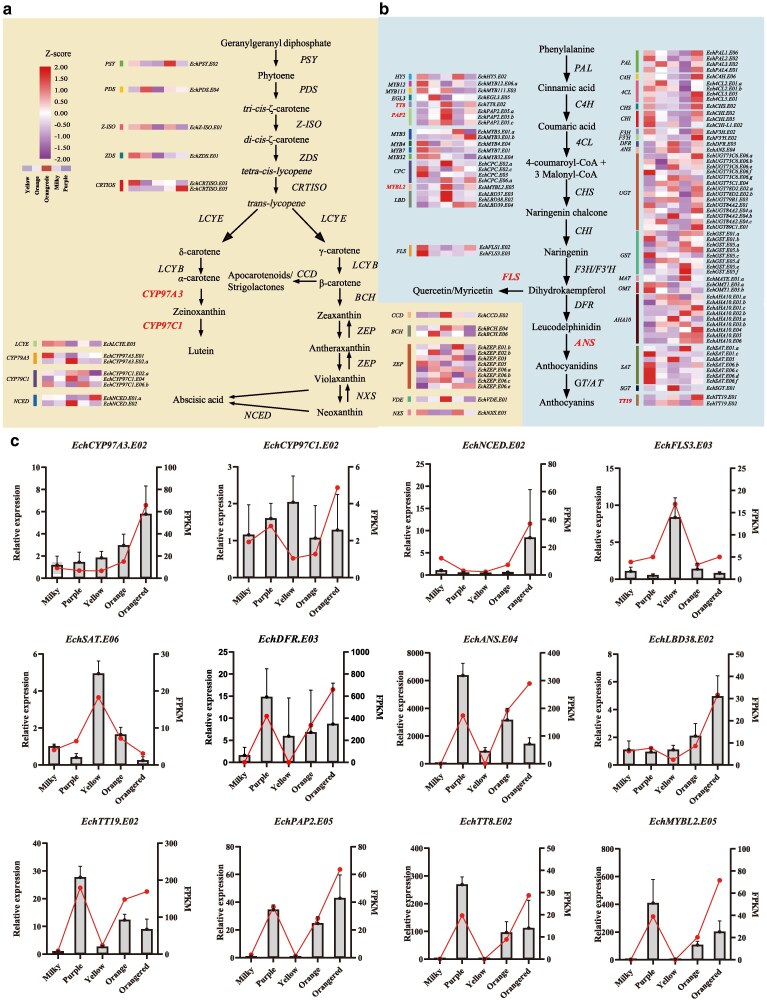
Carotenoid and flavonoid biosynthetic pathways in *E. cheiri*. **a)** Carotenoid and **b)** flavonoid pathway and related gene expressions in *E. cheiri*. The heatmaps indicate the gene expression levels in plants with different flower colors (yellow, orange, orangered, milky, and purple). The genes marked in red and bold are considered to be key gene groups regulating the synthesis of carotenoids, flavonoids and anthocyanins. Detailed information and data can be found in Tables S22–23. **c)** Relative expression levels of carotenoid and flavonoid pathway genes in *E. cheiri*. The gray bar graph is the result of RT-qPCR analysis, and the red line is the FPKM (fragments per kilobase of transcript per million mapped reads) value of RNA-seq analysis. The data are presented as the mean values of 3 biological replicates SD.

The flower color of *E. cheiri* is primarily determined by the biosynthesis and accumulation of different carotenoid and flavonoid compounds. To further investigate the expression patterns of carotenoid and flavonoid metabolic pathways and identify the key genes that regulate the synthesis of these metabolites, we identified the genes associated with carotenoid and flavonoid biosynthetic pathways in *E. cheiri* (Tables S22–23). Our results suggest that the genes in these 2 metabolic pathways were amplified to different extents in the genome of *E. cheiri*, particularly *CYP97C*1, *ZEP*, and *NCED* in the carotenoid biosynthetic pathway, and *PAP1/2*, *UGT73C6*, *SAT*, and *GST* in the flavonoid biosynthetic pathway. These gene amplifications may reflect natural selection during the evolution of *E. cheiri* and serve as the basis for the diverse color variants. We then integrated RNA-seq analysis to obtain expression data and constructed an expression heat map (Figs. 6a and b; Tables S22–23). Overall, the relative expression of carotenoid metabolic pathway genes was higher in yellow and orangered flowers, which had higher levels of carotenoid metabolites (Fig. 6a). In contrast, the expression of genes related to the flavonoid metabolic pathway was upregulated in yellow, orange, purple, and orangered flowers, that had higher flavonol or anthocyanin content (Fig. 6b).

KEGG enrichment analysis of TD-type genes revealed that these genes were specifically enriched in several metabolic pathways, including phenylpropanoid biosynthesis, the biosynthesis of various secondary metabolites, and anthocyanin biosynthesis. We analyzed the expansion of flavonoid biosynthesis genes (FBGs) and carotenoid biosynthesis genes (CBGs) in *E. cheiri*, categorizing them as either expanded genes or as TD-type or PD-type repetitive genes. Our findings indicate that 22 out of 118 FBGs were classified as TD-type repetitive genes, all of which were determined to be expanded genes. Additionally, 19 FBGs were identified as PD-type repetitive genes, with 5 of these also classified as expanded genes (Table S24). Among the 43 CBGs, only 4 were found to be TD-type repetitive genes, all of which were expanded; no PD-type repetitive genes were identified (Table S25). Furthermore, we identified 22 genes associated with the TD-type flavonoid biosynthetic pathway, 9 of which are located on fusion chromosomes E01 and E02 (Table S24). Among these 9 genes, 5 were expressed in the petals of *E. cheiri*. However, because we lacked comparable expression data from *E. cheiranthoides*, we were unable to conduct a comprehensive analysis of the expression pattern changes of these genes before and after the chromosome fusion events.

Then, we selected 12 DEGs from these 2 pathways, designed specific RT-qPCR markers, and conducted RT-qPCR analysis in petals of milky, purple, yellow, orange, and oranged flowers (Table S26). The results showed that the overall expression trends of *EchCYP97A3.E02*, *EchCYP97C1.E02*, and *EchNCED.E02* in the carotenoid metabolic pathway were consistent with the RNA-seq analysis, with higher expression levels in flower colors with higher carotenoid content (Fig. 6c). Notably, *EchFLS3.E03* and *EchSAT.E06*, the key genes involved in the synthesis of flavonols, showed higher expression levels in yellow flowers compared to other colors, consistent with the observation that yellow flowers possess the highest flavonol content (Fig. 6c). The 8 genes, *EchDFR.E03*, *EchANS.E04*, *EchUGT78D2.E02*, *EchLBD38.E02*, *EchTT19.E02*, *EchPAP2.E05*, *EchTT8.E02*, and *EchMYBL2.E05,* associated with the regulation of anthocyanin synthesis, were generally highly expressed in purple, orange, and orangered flowers. This expression pattern is a primary factor contributing to the extensive synthesis and accumulation of anthocyanins in these flower color variants (Fig. 6c).

## Discussion

Comprehensive and accurately annotated reference genomes are critical for research on genome evolution, functional gene discovery, genetic improvement, and breeding applications. *E. cheiri* is a plant species valued for its ornamental and medicinal properties. The previously assembled scaffold-level genome assembly of *E. cheiri* (Kiefer et al. 2019) was primarily utilized for phylogenetic and evolutionary studies within the Brassicaceae. In this study, PacBio HiFi, Hi-C, and Illumina sequencing data were used to assemble the nearly gap-free, high-quality genome of *E. cheiri*, representing a T2T genome for the genus *Erysimum*. This assembly demonstrates high contiguity of contigs and scaffolds, along with significant completeness of protein-coding genes and repeat sequences, providing a robust resource for genetic and evolutionary research on *E. cheiri* and related species.

The genus *Erysimum* exhibits considerable karyological variation at both the diploid and polyploid levels. Diploid chromosome numbers range from *n* = 6 to *n* = 28 (Warwick and Al-Shehbaz 2006). Comparison of the available chromosome-level genome assemblies, specifically the *n* = 8 genome of *E. cheiranthoides* (Zhai et al. 2024) and the *n* = 6 genome of *E. cheiri* (this study), provides only limited insight into the karyotype evolution in *Erysimum*. As the *n* = 7 genome of *E. nevadense* (Zhai et al. 2024) was not assembled to the chromosome level, its utility for reconstructing the process of descending dysploidy in *Erysimum* is limited. However, evidence suggests that *E. cheiri* and *E. nevadense* share the fusion chromosome 5/6 and that the origin of the fusion chromosome likely predated the later speciation of *Erysimum* species with *n* = 7 (6) chromosomes. The formation of the fusion chromosome E02 in *E. cheiri* superficially aligns with the definition of NCF, in which terminal regions of 1 chromosome recombine with (peri)centromeric repeats of a recipient chromosome, and the centromere of the inserted chromosomes remains functional (Luo et al. 2009). However, a detailed analysis of chromosome E02 revealed an inverse orientation of the genomic blocks (chromosome arms) of the inserted chromosome (chr 5), suggesting a more complex origin of the fusion chromosome (Fig. 2g). The NCF with a donor arm repositioning (Fig. 3b in Schubert and Lysak 2011) was described as mediating the origin of chromosome 4D in *Aegilops tauschii* (Luo et al. 2009). This type of NCF can lead to the origin of a hybrid centromere when recombination occurs between breaks in the centromeres of the participating chromosomes (Schubert and Lysak 2011; Lysak et al. 2016; Mandáková et al. 2017; Lysak 2022). However, the clear evidence for the hybrid origin of the E02 centromere is lacking. For instance, the length of the E02 centromere is comparable to that of the nonfusion chromosome E05 (Fig. S12). The second fusion chromosome, E01, is the longest chromosome in *E. cheiri*. This chromosome was formed by an end-to-end translocation between ancestral chromosomes 1 and 3 in a progenitor genome with *n* = 7 (Fig. 2h). It remains to be determined whether the descending dysploidy from *n* = 7 to *n* = 6 in other *Erysimum* species with *n* = 6 was caused by end-to-end translocations between the same chromosomes. The CCDB database (Rice et al. 2015; accessed on 5 September 2025) includes 8 species of *Erysimum*, including *E. cheiri*, with *n* = 6 chromosomes. Notably, all of these species are geographically confined to southeastern Europe, especially Greece and its neighboring regions (POWO, 2025). Phylogenetically, some species form monophyletic subclades, including *E. naxense*, *E. rhodium*, and *E. senoneri* (Moazzeni et al. 2014), as well as *E. cheiri* and *E. naxense* (Züst et al. 2020). Thus, we speculate that the fusion chromosome E01 is likely shared by a group of closely related *n* = 6 *Erysimum* species confined to southeastern Europe, rather than being specific to *E. cheiri*.

*E. cheiri*, an important ornamental plant, is cultivated globally due to its wide range of flower colors and remarkable stress resistance. The variety in flower colors is attributed to the accumulation of different types of carotenoids and flavonoids (Tanaka et al. 2008). Flavonoids are water-soluble pigments primarily found in the vacuoles, while carotenoids are fat-soluble pigments located in the chloroplasts and plastids of plants. These 2 types of pigments can coexist, adding to the color diversity in ornamental plants like *Chrysanthemum* (*Chrysanthemum morifolium*; Su et al. 2019), water lilies (*Nymphaea* spp.; Zhang et al. 2020), and roses (*Rosa chinensis*; Cheng et al. 2025). Interestingly, our research revealed that *E. cheiri* has maintained both carotenoid and flavonoid biosynthetic pathways, providing a genetic basis for the diverse variations in flower color. Notably, our repeated gene analysis has shown that PD- and TD-type duplicated genes experience stronger selective pressure, with these genes being notably enriched in the phenylpropanoid, flavonoid, and anthocyanin metabolic pathways. We suggest that this enrichment may result from adaptive evolution and extended artificial selection as an ornamental plant. While previous studies have used transcriptomic and metabolomic analyses to investigate flower color in *E. cheiri* (Chen et al. 2018; Tatsuzawa 2019), the lack of genomic information has limited these investigations. Here, we conducted a metabolomic analysis of petals, showing that variations in the composition and content of carotenoids, flavonols, and anthocyanins are the primary factors influencing differences in flower color. This finding has important implications for the cultivation of colorful wallflowers, as it can enhance their ornamental value and economic benefits.

Carotenoids, including α-carotene, β-carotene, and lutein, play a critical role in forming the yellow background of flowers, encompassing yellow, orange, and orangered hues. In contrast, the accumulation of anthocyanins causes a shift in flower color toward orange and orangey-red tones. This color change is due to the interaction between carotenoid and anthocyanin metabolites, resulting in a co-coloring effect. Flowers that appear milky exhibit the lowest levels of carotenoid and flavonoid metabolites, leading to their lighter coloration. Previous studies suggest that this phenomenon may be linked to the degradation of carotene by CAROTENOID CLEAVAGE DIOXYGENASE 4 (Zhang et al. 2015; Amaya et al. 2025). The buildup of anthocyanins changes the flower color from milky to purple. Our RNA-seq and RT-qPCR analyses indicate that genes *EchCYP97A3.E02*, *EchCYP97C1.E02*, and *EchNCED.E02* are crucial for the synthesis and accumulation of carotenoids in *E. cheiri*, while *EchFLS3.E03* and *EchSAT.E06* primarily facilitate the synthesis of flavonols. Additionally, genes *EchANS.E04*, *EchUGT78D2.E02*, *EchLBD38.E02*, *EchTT19.E02*, *EchPAP2.E05*, *EchTT8.E02*, and *EchMYBL2.E05* mainly regulate the synthesis and accumulation of anthocyanins, which aligns with our previous findings (Chen *et al.* 2018). The results of this study provide a foundation for further exploration of the molecular mechanisms underlying flower color variation in *E. cheiri*. In our previous research, we successfully transferred *OvPAP2*, a key transcription factor that regulates flower color from *O. violaceus*, into canola (*B. napus*). This resulted in the creation of a novel variety characterized by red flowers (Fu et al. 2018). The carotenoid and anthocyanin regulatory genes identified in the present study may not only serve as targets for molecular breeding in wallflower but also provide valuable gene sources for enhancing flower color in species within the Brassicaceae family. Moreover, *E. cheiri* has a relatively simple genome and can alternate between annual and perennial flowering and fruiting behaviors (Zhai et al. 2024). This unique trait makes it an excellent model for studying the molecular mechanisms of flower color variation in plants and suggests it could be a valuable candidate for research aimed at genetically enhancing flower color.

In summary, this study presents a high-quality T2T genome assembly of *Erysimum cheiri* and the tribe Erysimeae. We reconstructed the origin of the *E. cheiri* genome by examining the inferred ancestral karyotypes of the supertribe Camelinodae. Additionally, we analyzed the fusion events that transformed the 8 ancestral *Erysimum* chromosomes into complements with 7 and 6 chromosomes. The comprehensive genome and gene annotations provide a solid foundation for understanding the biosynthesis of carotenoids and flavonoids. They also help identify candidate structural genes and transcription factors that regulate the production of carotenoids, and flavonoids. Moreover, this research offers valuable resources for exploring the molecular mechanisms behind important agronomic traits, such as linoleic acid metabolism, cardenolide synthesis, and strong stress resistance, providing a foundation for future genetic improvement and molecular breeding of *E. cheiri*.

## Materials and methods

### Plant material

The *E. cheiri* seeds used in this study were provided by X. Ge (Huazhong Agricultural University, Wuhan, China). All plants were grown from seeds and cultivated in a greenhouse at Gannan Normal University, Ganzhou, China. After 8 generations of selection, 5 stable inbred lines with different flower colors were obtained: (i) yellow, (ii) orange, (iii) orangered, (iv) milky, and (v) purple. The seeds can be obtained from the authors on request.

### Sample preparation, library construction, and sequencing

Fresh and healthy leaves were collected from 2-mo-old *E. cheiri* plants and immediately frozen in liquid nitrogen. The extracted genomic DNA was evaluated for quality and quantity using the NanoDrop One spectrophotometer and the Qubit 3.0 fluorometer. The qualified DNA was then used to construct NGS libraries and subjected to 350 bp paired-end sequencing on the DNBSEQ-T7 platform. For PacBio HiFi sequencing, the SMRTbell Express Template Prep Kit 2.0 (Pacific Biosciences) was used to construct libraries and perform single-molecule sequencing on the Sequel II system. In addition, 2-mo-old young leaves of *E. cheiri* were fixed with 1% (v/v) formaldehyde and used for Hi-C library construction. This process included cross-linking, chromatin quadruple digestion with Mbo I, DNA end labeling, ligation purification, shearing, and biotin pull-down. Subsequently, the Hi-C library was sequenced using a NovaSeq 6,000 sequencer in 150 bp paired-end mode. Seedlings (7 days after germination), leaves (2 mo old), roots (2 mo old), stems (during the bolting period), inflorescences, young siliques (3 weeks after blooming), and mature seeds were collected, immediately frozen in liquid nitrogen and stored at −80 °C for transcriptome sequencing to aid in whole-genome annotation.

### Genome survey

The genome size of *E. cheiri* was initially estimated using *k-mer* analysis. Approximately 37.05 Gb of filtered clean reads were used to calculate the 21-mer frequency distribution using Jellyfish v2.2.10 (Marçais and Kingsford 2011). Genome size and heterozygosity were computed using the GenomeScope v2.0 (Vurture et al. 2017).

### Genome assembly and assessment

To assemble the *E. cheiri* genome de novo, we used the default parameters of the hifiasm software v0.16.1 (Cheng et al. 2021) to construct draft contig genome assemblies using PacBio HiFi data. The main contigs were then adjusted for subsequent analysis. The assembly was polished using minimap2 v2.17 (Li, 2018) and Racon v1.4.19 (Vaser et al. 2017), which utilized long reads. Additionally, possible sequencing and assembly errors were corrected using BWA v0.7.17 (Li and Durbin 2009) and Pilon v1.23 (Walker et al. 2014) were used with Illumina short-read data. The pseudo-chromosomes were constructed using Hi-C data through the Juicer v2.0 (Durand et al. 2016) and 3D-DNA v180922 (Dudchenko et al. 2017). The default parameters were used for grouping, ordering, and orienting the contigs. The Juciertools software v3.0 (Durand et al. 2016) was used to convert the interactions between the contigs into a specified binary file format (ie, .hic file). The sequenced and oriented contigs were manually corrected using Juciebox software v2.15.07 (Robinson et al. 2018) to obtain the final chromosome-level assembly results. To examine the integrity of the assembly, the long reads were aligned to the assembly using minimap2, while short reads were aligned using BWA. RNA-seq data were also aligned back to the genome to verify the quality of the genome assembly using HISAT2 v2.1.0 (Kim et al. 2019). The quality of the assembled genome was assessed using BUSCO v5.6.1 (Simão et al. 2015). LTR_FINDER v1.07 (Xu and Wang 2007) and LTRharvest v1.5.10 (Ellinghaus et al. 2008) were used to identify and annotate long terminal repeats (LTRs) in the assembly. These results were then integrated to calculate the LAI using LTR_retriever v2.9.0 (Ou et al. 2018; Ou and Jiang 2018).

### Identification of telomeres and centromeres

The quarTeT toolkit v1.2.4 (Lin et al. 2023) was used to predict telomere and centromere regions. Telomeres were predicted using the TeloExplorer module, while centromeres were predicted using the CentroMiner module. The Hi-C interaction heatmaps were also used to identify centromere and telomeric regions.

### Genome annotation

We used RepeatModeler2 v4.1.9 (Flynn et al. 2020) to predict the repetitive sequences of the genome from scratch (de novo). These predicted results were then merged with the RepBase database (http://www.girinst.org/repbase/). Following this, we employed RepeatMasker v4.1.0 (Burkes-Patton et al. 2023) and the EDTA pipeline v2.2.2 (Ou et al. 2019) for further prediction of repetitive sequences in the genome; and the annotation results of EDTA pipeline were used for subsequent analysis. For mRNA prediction, we used 3 methods: de novo prediction, homology prediction, and transcript prediction. The de novo prediction was conducted using Augustus v3.3.3 (Stanke et al. 2004) and GlimmerHMM v3.0.4 (Majoros et al. 2004), with models trained on a set of high-quality proteins generated from the ISO-seq dataset. We downloaded reference protein sequences of *Arabidopsis thaliana* (Col-XJTU, TAIR10), *B. rapa* (Chiifu), *B. napus* (ZS11 v10), *B. juncea* (Varuna), and *Raphanus sativus* (NAU-LB) to facilitate homologous gene predictions (Table S6). Homology-based gene prediction was performed using GeMoMa software v1.9 (Keilwagen et al. 2019). For transcript reconstruction, RNA-seq data were analyzed using StringTie v2.1.3 (Pertea et al. 2015). Then, TransDecoder v5.1.0 (https://github.com/TransDecoder/) was used to predict coding frames. The multiple datasets obtained were integrated using EvidenceModeler v1.1.1 (Haas et al. 2008). Finally, we used PASA pipeline v2.5.2 (Haas et al. 2008) to update the integrated data, add UTR regions, and identify new transcripts.

The prediction of non-coding RNAs included 3 components: (i) Using barrnap v0.9 (https://github.com/tseemann/barrnap) to predict rRNA sequences. (ii) Using tRNASCAN v2.0.0 (Chan et al. 2021) to predict tRNA sequences. (iii) Using Infernal v1.1.3 (https://github.com/EddyRivasLab/infernal) to retrieve data from the Rfam database (https://rfam.org/) or non-coding RNA predictions.

Gene functions were inferred by aligning sequences to the National Center for Biotechnology Information (NCBI) Non-Redundant and Uniprot (Swiss-Prot and TrEMBL) protein databases, using Diamond v2.0.7.145 (Buchfink et al. 2015). The predicted protein sequences were then uploaded to KAAS (Moriya et al. 2007) to obtain the Kyoto Encyclopedia of Genes and Genomes (KEGG) annotation, and using eggNOG (Huerta-Cepas et al. 2019) for additional information. Protein domains were annotated using HMMER v3.3.1 (Mistry et al. 2013) based on Pfam databases. Gene Ontology (GO) annotations for each gene were obtained from InterProScan software v5.50 to 84.0 (Zdobnov and Apweiler 2001). The methods and processes for genome annotation are consistent with those previously described (Chen et al. 2025).

### Genomic evolutionary analysis

Transposable elements (TEs) in the genome of *E. cheiri* were identified using the EDTA pipeline. The results regarding TE divergence and the insertion times of long terminal repeat retrotransposons (LTR-RTs) were extracted from the intermediate files generated by EDTA. Unknown types of LTR-RTs, identified by EDTA, were accurately classified using DeepTE software v3.7.1 (Yan et al. 2020). To identify common gene families among the species *Ae. arabicum*, *Ar. alpina*, *A. lyrata*, *B. rapa*, *B. nigra*, *E. cheiranthoides*, *E. nevadense*, *L. alabamica*, *M. incana*, *O. violaceus*, and *R. sativus*, we used OrthoFinder software v2.5.2 (Emms and Kelly 2019) along with Diamond (Table S9). Based on the protein sequences of 819 single-copy orthologous families, we aligned the protein sequences and their corresponding coding DNA sequences using MUSCLE v3.8.31 (Edgar 2004). The phylogenetic relationships among these species were estimated with RAxML software v8.2.12 (Stamatakis 2014) using a maximum likelihood method. The time correction points were obtained from the TimeTree website (http://www.timetree.org/), the calibration time points for estimating divergence time were 4.4 million years ago (Mya) < (*E. cheiri*, *E. nevadense*) < 5.0 Mya, 3.9 Mya < (*A. lyrata*, *A. thaliana*) < 16.3 Mya, 4.5 Mya < (*B. rapa*, *B. nigra*) < 11 Mya, 8.6 Mya < (*R. sativus*, *B. rapa*) < 24.7 Mya. Divergence times were estimated using the paml v4.9 MCMCTree program (Yang 2007) with the following parameters: burn-in & 10,000, sample size & 50,000, and sample frequency & 10.

### Comparative genomic analysis

We used CAFE v4.2.1 (Lu et al. 2017) software to analyze gene family contraction and expansion. Families with more than 100 genes were chosen for the analysis, and a *P-*value threshold of 0.01 was selected as the standard. Next, we examined the occurrence of whole-genome duplications (WGDs) in *E. cheiri*. We conducted an all-to-all search in Diamond, with an e-value set at 1e-5, to identify all homologous proteins between *E. cheiri* and 5 other species (*Ar. alpina*, *A. thaliana*, *Ae. arabicum*, *B. rapa*, and *E. cheiranthoides*). The WGDI software version 0.4.7 (Sun et al. 2022) was used to extract collinear blocks, estimate nonsynonymous (*Ka*) and synonymous (*Ks*) substitution rates, and generate a distribution map of *Ks* values.

For the collinearity comparison, we used JCVI software v1.5.6 (Tang et al. 2024). To align the amino acid sequences of paired genes identified in the MCScanX results (Wang et al. 2012). The aligned amino acid sequences were subsequently converted into nucleic acid sequences. To classify and identify genomic gene duplications, we used DupGen-finder (https://github.com/qiao-xin/DupGen_finder) with blastp parameters set to an e-value of 1e-10 and -max_target_seqs of 10. The *Ka*/*Ks* values of the genes were calculated using KaKs_Calculator software v3.0 (Zhang 2022), with the YN method selected for these calculations. GO and KEGG enrichment analyses were performed using the R package clusterProfiler v4.3.0 (Yu et al. 2012).

### rDNA FISH and comparative chromosome painting

Mitotic and meiotic chromosome spreads were prepared according to Mandáková and Lysak (2016a). For comparative chromosome painting (CCP), *A. thaliana* BAC clones were grouped into contigs to identify the 22 conserved genomic blocks of Brassicaceae genomes (see Mandáková et al. 2019 for the limits of these genomic blocks). The clone pCT 4.2, corresponding to a 500-bp 5S rRNA repeat (M65137), was used for the localization of 5S rDNA loci, while the *A. thaliana* BAC clone T15P10 (AF167571) bearing 35S rRNA genes was used for the localization of nucleolar organizer regions.

Nick translation was used to label all DNA probes with either biotin-dUTP, digoxigenin-dUTP, or Cy3-dUTP, which were then pooled and precipitated. The DNA:DNA in situ hybridization and post-hybridization detection of the hybridized DNA probes followed the published protocol (Mandáková and Lysak 2016b). Digoxigenin-dUTPs were identified using mouse antidigoxigenin (Jackson ImmunoResearch) and goat anti-mouse Alexa Fluor 488 (Invitrogen), while biotin-dUTP was detected using avidin∼Texas Red (Vector Laboratories) and ampliﬁed by goat anti-avidin∼biotin (Vector Laboratories) and avidin∼Texas Red. Chromosomes were counterstained with 4′,6-diamidino-2-phenylindole (2 mg/mL) in Vectashield (Vector Laboratories). An AxioImager epifluorescence microscope (Zeiss) and a CoolCube camera (MetaSystems) were used to capture and photograph fluorescent signals. The captured images were pseudocolored and merged using Adobe Photoshop CS6 software (Adobe Systems).

### Detection of flavonoid and carotenoid metabolites

The carotenoid and flavonoid metabolites were detected by MetWare Biological Co., Ltd (Wuhan, China). The petals of the freshly opened buds were collected, immediately frozen with liquid nitrogen, and stored at −80 °C for metabolome analysis. Carotenoid and flavonoid contents were determined using MetWare (http://www.metware.cn/) based on the AB Sciex QTRAP 6,500 LC-MS/MS platform. Flower samples of 5 colors, with 3 biological replicates each, were freeze-dried, ground into a powder (30 Hz, 1.5 min), and stored at −80 °C. For extraction, 50 mg of the powder was mixed with 0.5 mL of a methanol/water/hydrochloric acid solution (500:500:1, v/v/v), followed by vortexing (5 min), ultrasound treatment (5 min), and centrifugation at 12,000 g for 3 min at 4 °C. The residue was re-extracted by repeating the same procedure. The supernatants were collected and filtered through a 0.22 μm membrane filter (Anpel) before LC-MS/MS analysis. The sample extracts were analyzed using a UPLC-ESI-MS/MS system (UPLC, ExionLC™ AD, https://sciex.com.cn/, MS Applied Biosystems 6,500 Triple Quadrupole, https://sciex.com.cn/). The analytical conditions were set as follows: for UPLC, a Waters ACQUITY BEH C18 column (1.7 µm, 2.1 mm × 100 mm) was used, with a solvent system consisting of water (0.1% formic acid) and methanol (0.1% formic acid). The gradient program began with a composition of 95:5 (v/v) at 0 min, shifted to 50:50 (v/v) at 6 min, changed to 5:95 (v/v) at 12 min, and held for 2 min before running to 95:5 (v/v) at 14 min, with another 2-min hold. The flow rate was 0.35 mL/min, the temperature was maintained at 40 °C, and the injection volume was 2 μL. Linear ion trap (LIT) and triple quadrupole (QQQ) scans were conducted using a QQQ-LIT mass spectrometer (QTRAP) model 6,500+ LC-MS/MS System. This system was equipped with an ESI Turbo Ion-Spray interface, operating in positive ion mode and controlled by Analyst 1.6.3 software (Sciex). The operation parameters for the ESI source were as follows: the ion source was set to ESI+; the source temperature was 550 °C; the ion spray voltage (IS) was 5,500 V; and the curtain gas was maintained at 35 psi. Anthocyanins were analyzed using scheduled multiple reaction monitoring (MRM). Data acquisition was performed with Analyst 1.6.3 software (Sciex), while Multiquant 3.0.3 software (Sciex) was used for quantifying all metabolites. The mass spectrometer parameters, including the declustering potentials and collision energies (CE) for individual MRM transitions, were further optimized for accuracy. A specific set of MRM transitions was monitored during each time period based on the metabolites eluting at that time. Differential metabolites (DEMs) between groups were determined by the absolute log_2_ Fold Change (log_2_ FC). The TBtools-II software v2.118 (Chen et al. 2023) was used to create a heat map of the carotenoid and flavonoid metabolites, highlighting their Log_2_FC value.

### RNA sequencing and transcriptome analysis

Petals from buds of plants with differently colored flowers, with 3 biological replicates, were extracted using Eastep Total Kit (Promega, Beijing, China) according to the manufacturer's protocol. Subsequently, total RNA was identified and quantified using a Qubit fluorescence quantifier and a Qsep400 high-throughput biofragment analyzer. To isolate cDNA fragments approximately 250 to 350 bp in length, the library fragments underwent purification using the AMPure XP system (Beckman Coulter, Beverly, CA, USA). DNA fragments with adaptor molecules ligated to both ends were selectively enriched using the Illumina PCR Primer Cocktail during a 15-cycle PCR. The resulting products were purified again using the AMPure XP system and quantified with the Agilent High Sensitivity DNA assay on a Bioanalyzer 2100 system (Agilent). Finally, the sequencing library was sequenced on the Illumina NovaSeq 6000 platform.

We used Trimmomatic software v0.39 (Bolger et al. 2014) to trim the paired-end reads, removing adaptors and low-quality reads. Additionally, we excluded reads with a trimmed length of less than 100 bp. Clean reads were then aligned to the de novo assembled genome using HISAT2 software v2.1.0 (Kim et al. 2019) with default settings. We calculated read counts and gene expression levels in FPKM (Fragments Per Kilobase Million) for each gene using StringTie based on gene length. TBtools-II software was used to create a heat map of gene expression levels related to the carotenoid and flavonoid biosynthesis pathways with *Z*-score.

For the analysis of significantly differentially expressed genes (DEGs) between 2 groups, we used DESeq2 v4.5 (Love et al. 2014); genes with a *P-*value ≤ 0.01 and a Log_2_FC ≥2 were considered DEGs. GO and KEGG enrichment analysis of DEGs was conducted using clusterProfiler v4.0 (Wu et al. 2021).

We performed weighted gene co-expression network analysis (WGCNA) following established protocols. Differentially abundant metabolites and DEGs were used to construct co-expression network modules using the WGCNA package in R. Modules related to flavonoids and carotenoids with the highest correlation coefficients were selected for further analysis. Transcriptional regulatory networks were established using Pearson's correlation |*r*| > 0.8 and *P-*value < 0.05 among key differential metabolites, structural genes, and transcription factors. Network visualization was performed using Cytoscape v3.10.4 (https://cytoscape.org/).

### Quantitative analysis of key genes in carotenoid and flavonoid metabolic pathways

Total RNA was isolated from petals of closed flower buds, with 3 biological replicates for each flower color variant. We then synthesized cDNA via reverse transcription quantitative PCR (RT-qPCR) analysis, following the protocol for ChamQ SYBR qPCR Master Mix (Q711-02) (High ROX Premixed, Vazyme). We used *Actin3* as the internal reference gene, and the relative gene expression was calculated according to the 2^−ΔΔ^CT method. Gene-specific primers were designed for carotenoid and flavonoid metabolic pathway genes are listed in Table S26. The detailed RT-qPCR experimental procedure and data analysis methods were described earlier (Chen et al. 2020; Tan et al. 2024).

## Supplementary Material

kiag133_Supplementary_Data

## Data Availability

The raw genome sequencing and transcriptome data reported in this study are accessible through NCBI under the accession number SAMN49991968 and PRJNA1289382. The genome assembly and annotation data can be found in the NCBI database with the BioProject identifier PRJNA1292142.
