# Supplementary material for: Insights into karyotype evolution and flower color variation from the genome assembly of wallflower (Erysimum cheiri)
Source: Plant Physiol. 2026 Mar 13;200(4):kiag133. doi: 10.1093/plphys/kiag133 (PMC13089509; doi:10.1093/plphys/kiag133)
Supplement: kiag133_Supplementary_Data [file kiag133_supplementary_data.zip › PLPHYS-2025-1733R2_Supplementary Figures.pdf]

# Supplementary Figures

## Insights into karyotype evolution and flower color variation from the genome assembly of wallflower (*Erysimum cheiri*)

Chen *et al.*

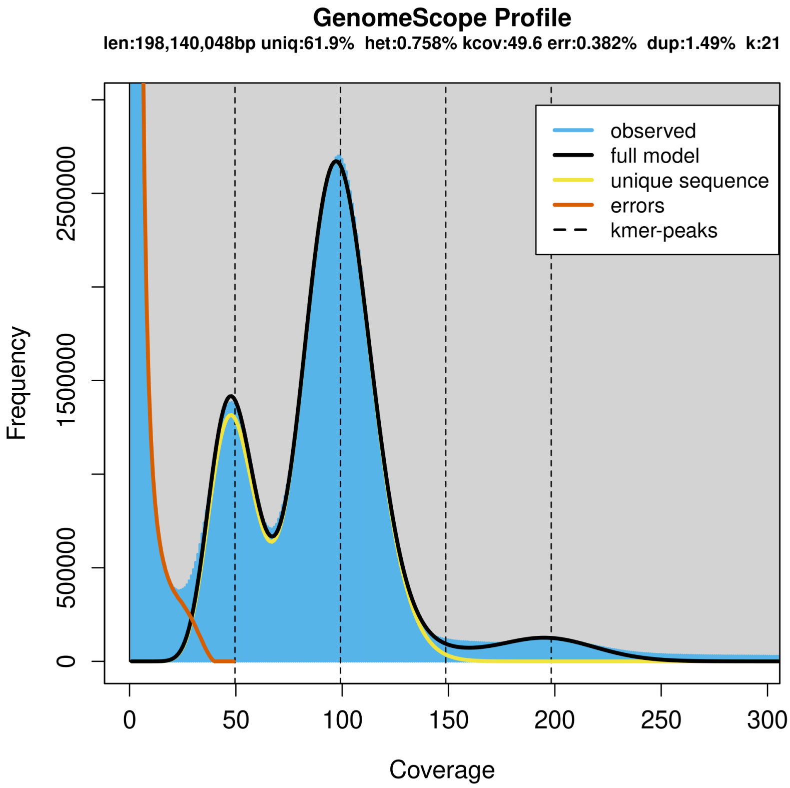


**Supplementary Figure S1 Analysis of the *E. cheiri* genome size conducted using *k-mer* analysis.** The *k-mer* frequency distribution ($k$ = 21) shows that the estimated genome size of *E. cheiri* is 198.14 Mb, with a heterozygosity rate of 0.78%.

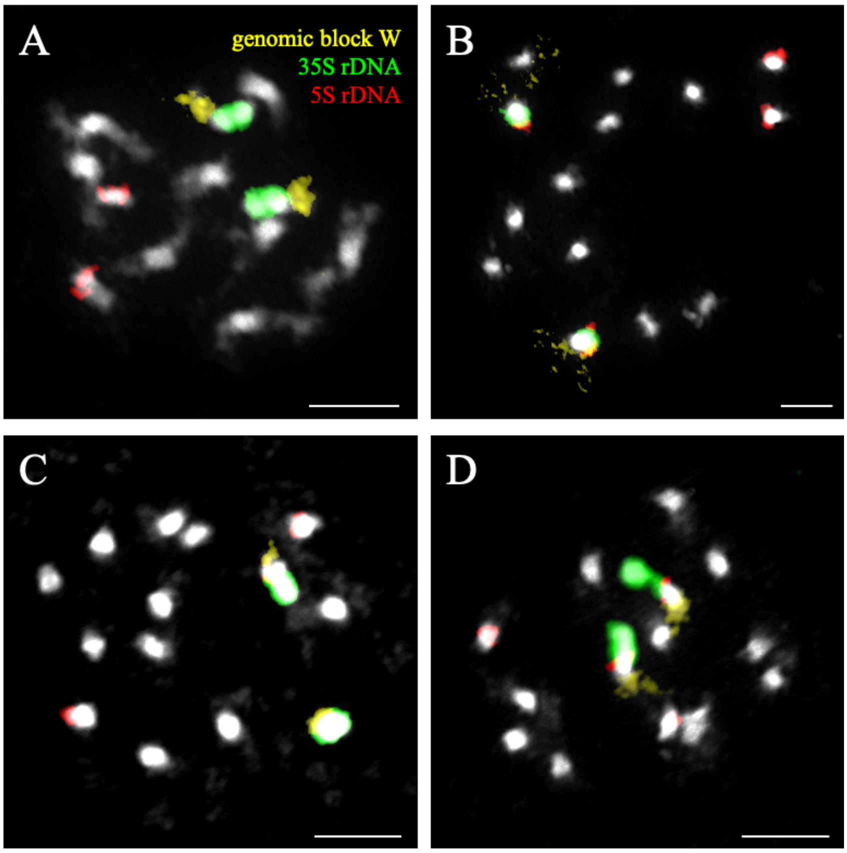


**Supplementary Figure S2. Chromosomal position of rDNA loci in selected *Erysimum* accessions/species. (A)** *E. cheiri* (2*n* = 12; Vacratot Botanical Garden, accession #955). **(B)** *E. crepidifolium* (2*n* = 14; Slovakia, Plástovce; 48.1654261N, 18.9834242E). **(C)** *E. odoratum* (2*n* = 14; Slovakia, Nitra; 48.3456658N, 18.1066039E). **(D)** *E. odoratum* (2*n* = 14; Slovakia, Plavecké Podhradie; 48.4864153N, 17.2636025E). Mitotic chromosomes were hybridized with 5S rDNA (red), 35S rDNA (green), and *Arabidopsis* BAC clones specific to genomic block W (yellow). Chromosomes were counterstained with DAPI. Scale bars,10 µm.

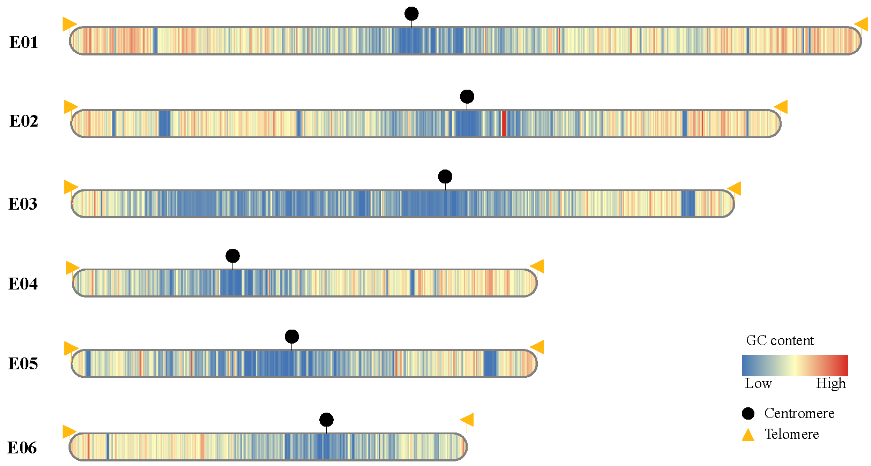


**Supplementary Figure S3 Location of telomeres and centromeres on *E. cheiri* chromosomes.**

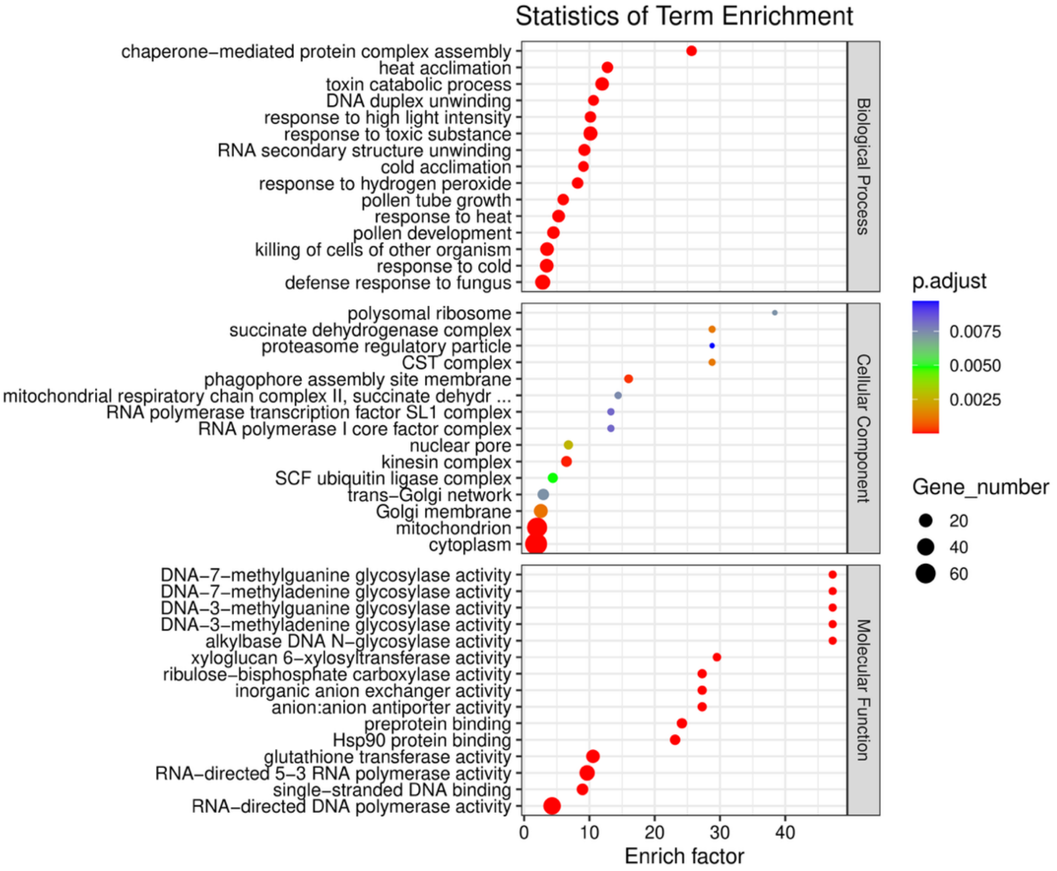


**Supplementary Figure S4 Gene Ontology (GO) enrichment analysis of the unique genes in *E. cheiri*.**

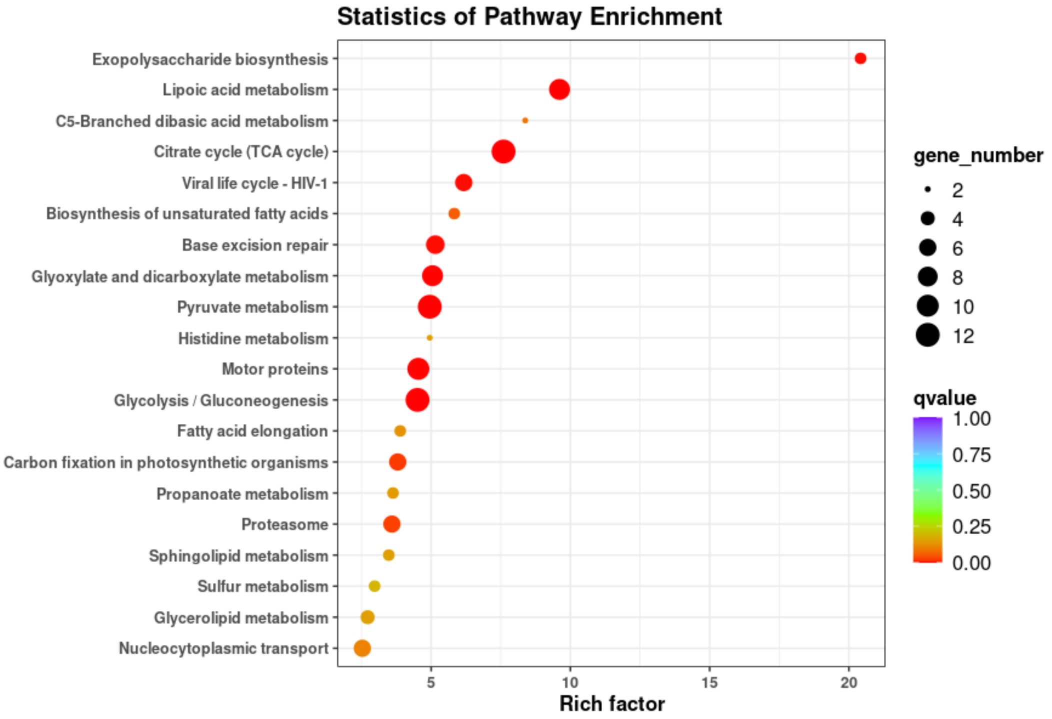


**Supplementary Figure S5 Kyoto Encyclopedia of Genes and Genomes (KEGG) enrichment analysis of the unique genes in *E. cheiri*.**

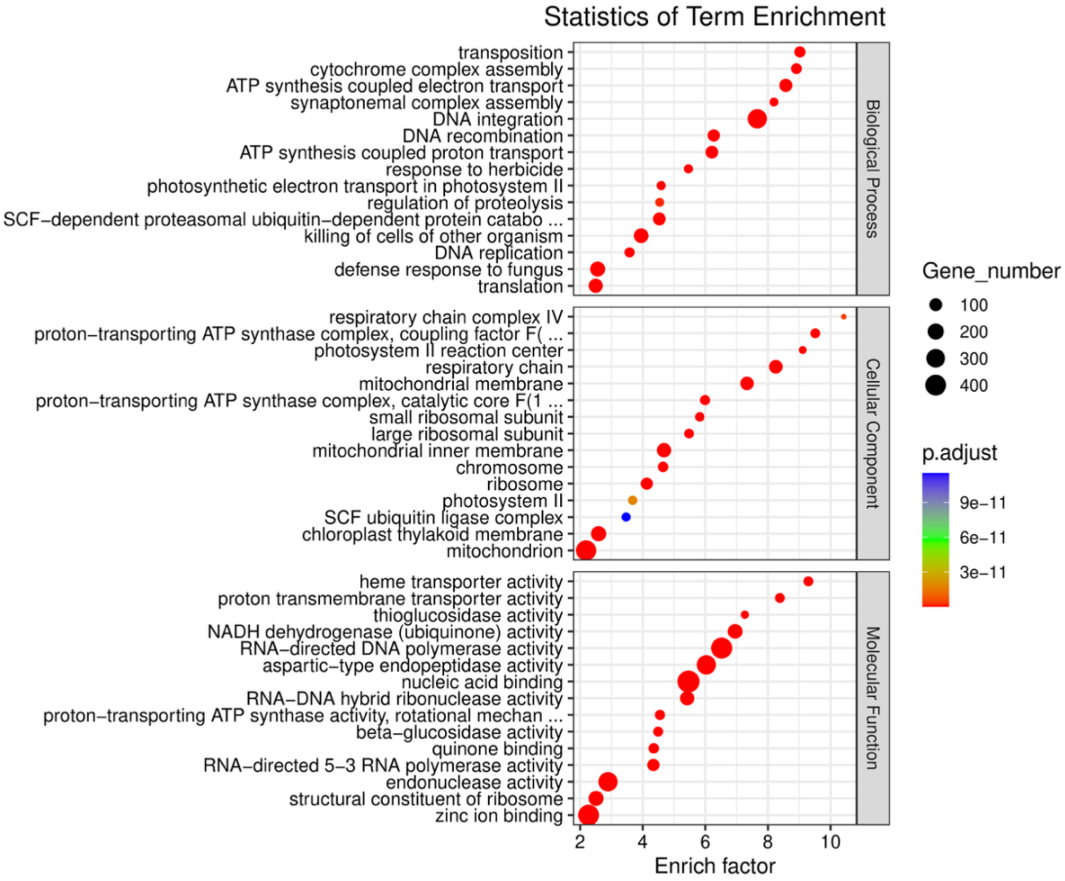


**Supplementary Figure S6 GO enrichment analysis of the expanded gene families in *E. cheiri*.**

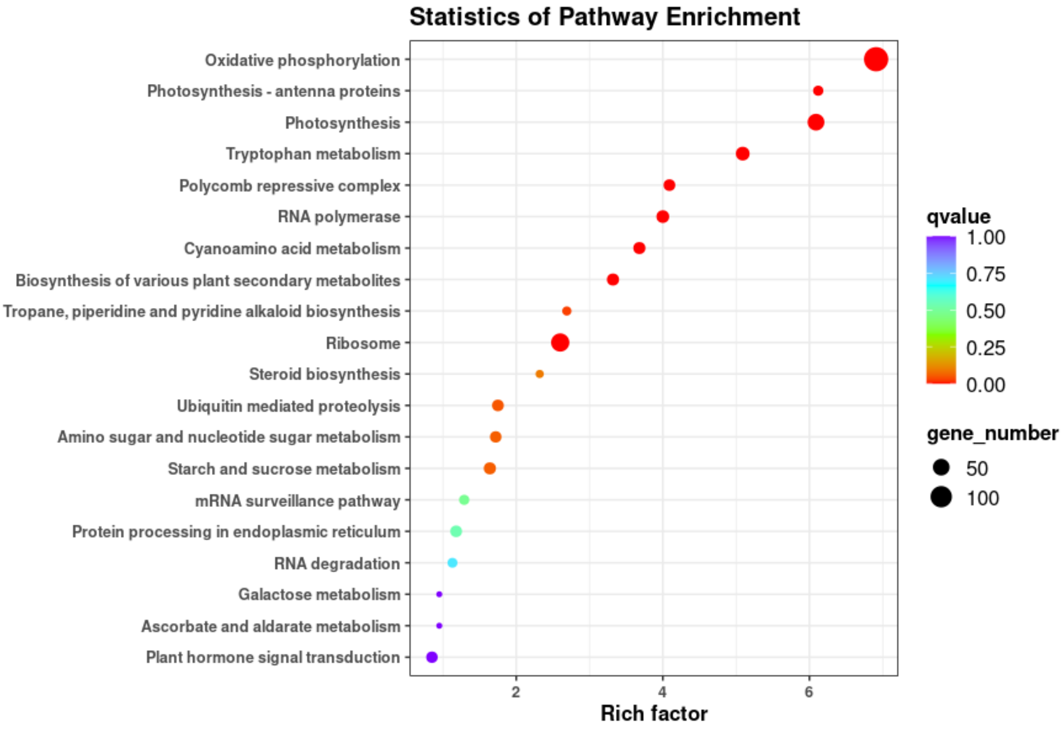


**Supplementary Figure S7 KEGG enrichment analysis of the expanded gene families in *E. cheiri*.**

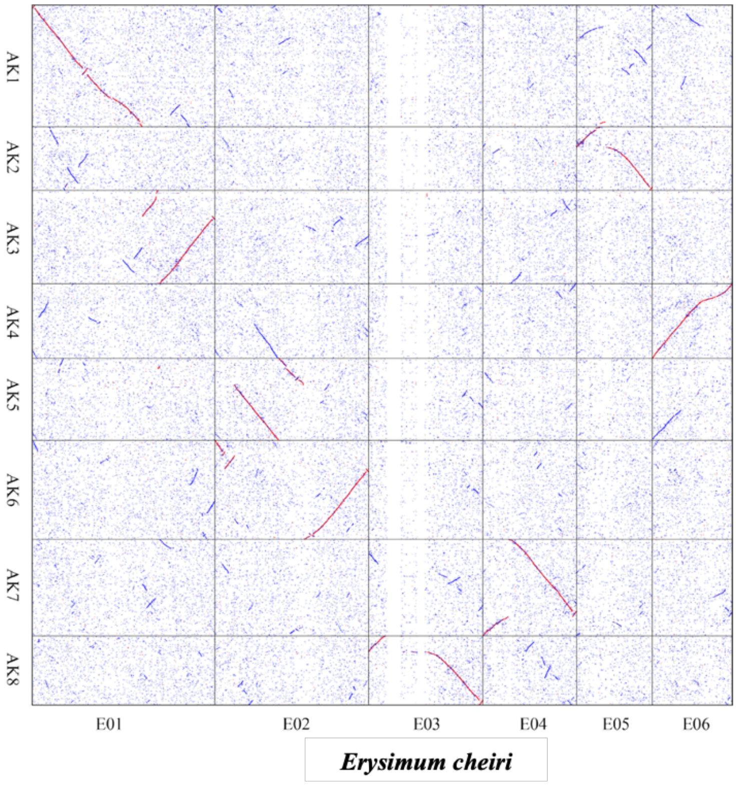


**Supplementary Figure S8 Collinearity analysis between *E. cheiri* and Ancestral Crucifer Karyotype (ACK, Lysak *et al*., 2016).** The *Arabidopsis thaliana* TAIR10 genome was used to reconstruct the eight AK chromosomes.

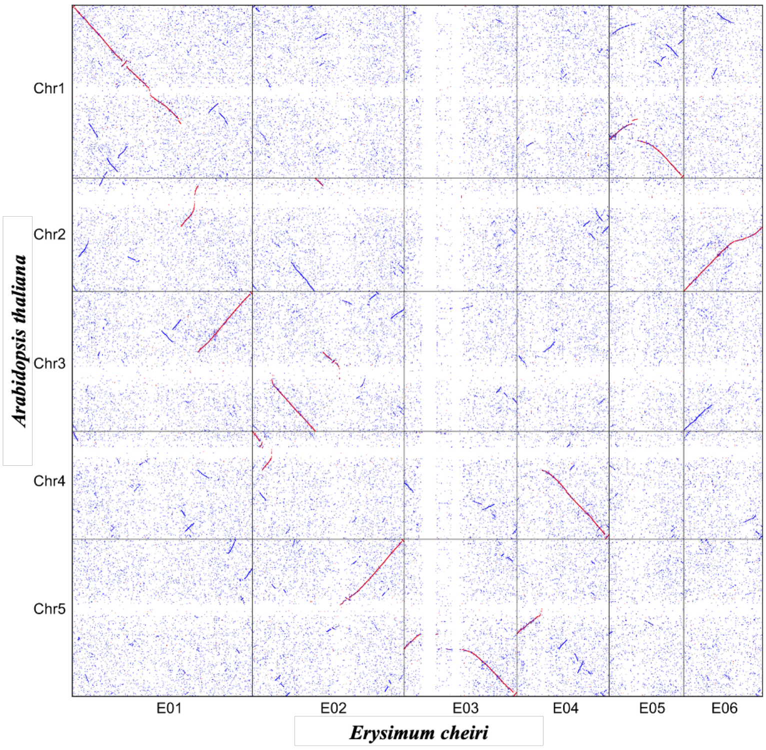


**Supplementary Figure S9 Collinearity analysis between chromosomes of *E. cheiri* and *A. thaliana* (TAIR10).**

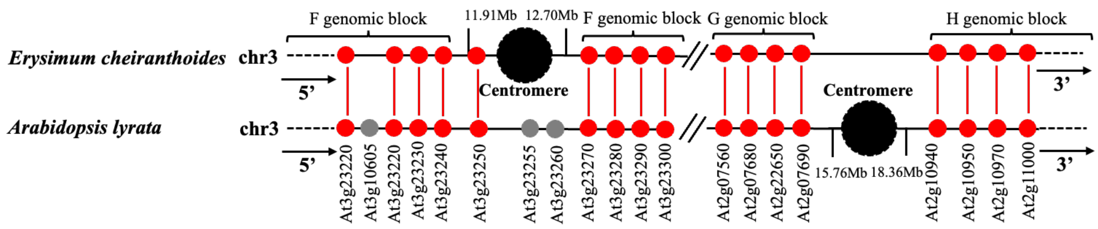


**Supplementary Figure S10 Diagram illustrating the centromere repositioning on chromosome chr3 in *E. cheiranthoides* (Zhai *et al*., 2024).** Chromosome 3 of *A. lyrata* (v2.1, http://brassicadb.cn/#/Download/) was used as a proxy for the purported ancestral homeologue of chromosome 3 in *E. cheiranthoides*.

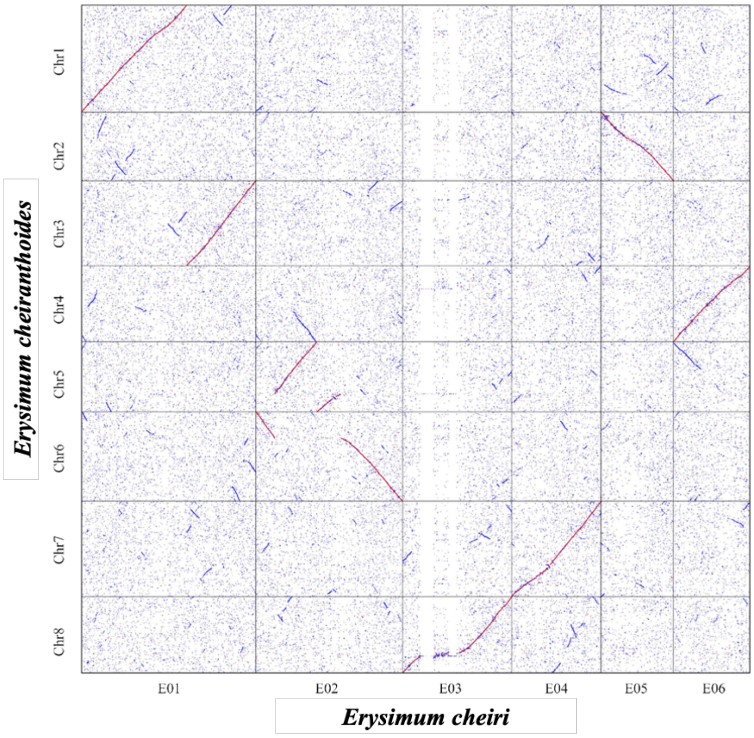


**Supplementary Figure S11 Collinearity analysis between chromosomes of *E. cheiri* and *E. cheiranthoides* (Zhai *et al*., 2024).**

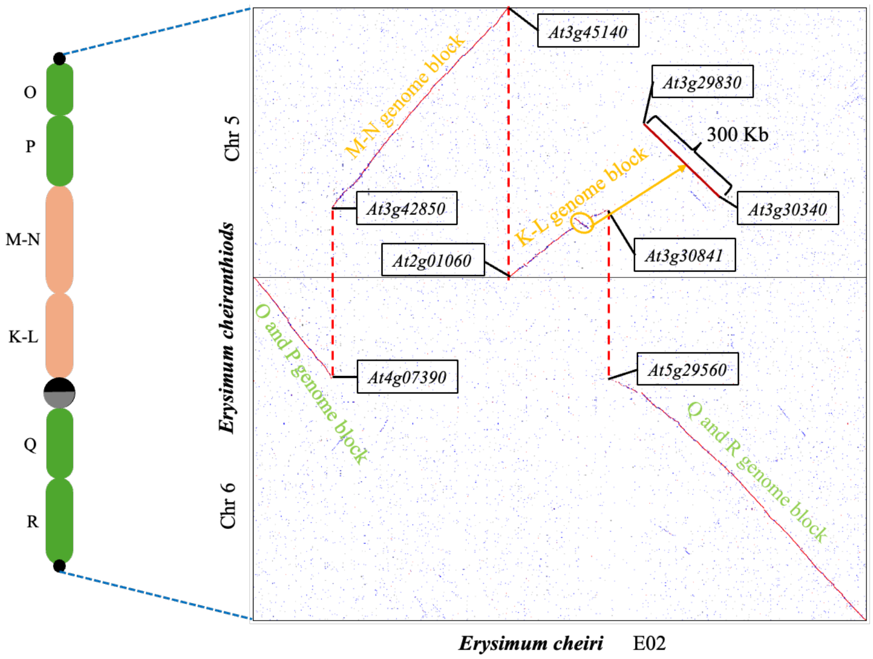


**Supplementary Figure S12 The detailed information on collinearity comparison between chromosome E02 of *E. cheiri* and chromosomes chr5 and chr6 of *E. cheiranthoides* (Zhai *et al*., 2024).** Capital letters represent genomic blocks of ACK (Lysak *et al.*, 2016). The zoomed-in inset shows the 300-kb inversion within the K-L block on E02. The *A. thaliana* gene names are the boundary genes of the homeologous genomic blocks.

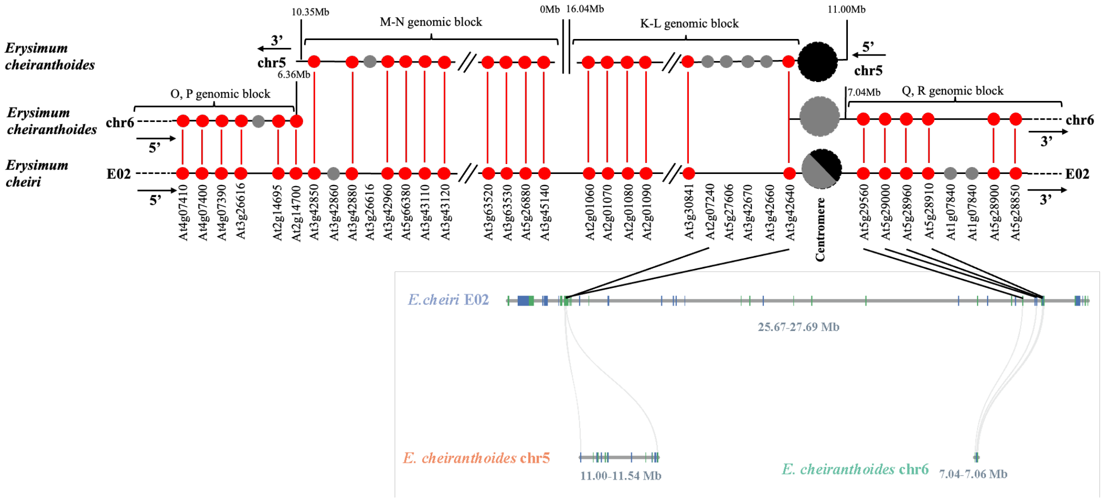


**Supplementary Figure S13 The detailed analysis of nested chromosome fusion (NCF) forming chromosome E02 in *E. cheiri*.** Homeologue of chr5 was "inserted" into the recipient chr6 homeologue of *E. cheiranthoides* (Zhai *et al*., 2024). Red dots and lines indicate syntenic genes, whereas gray dots indicate the missing homologous genes.

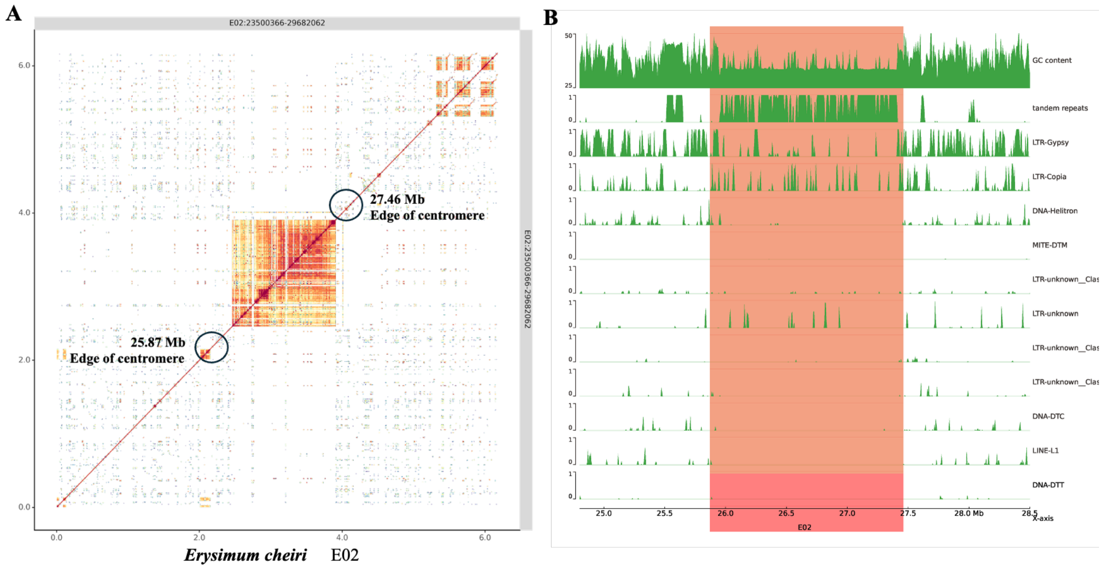


**Supplementary Figure S14 The distribution of repetitive sequences in the centromeric region of chromosome E02 in *E. cheiri*. (A)** The heatmap illustrates the repetitive sequences on E02 in *E. cheiri*. Black circles indicate the positions of homologous genes between *E. cheiranthoides* (Zhai *et al*., 2024) and *E. cheiri*. The E02 centromere spans the interval from 25.87 to 27.46 Mb. **(B)** GC content and distribution of various types of repetitive sequences within the E02 centromere.

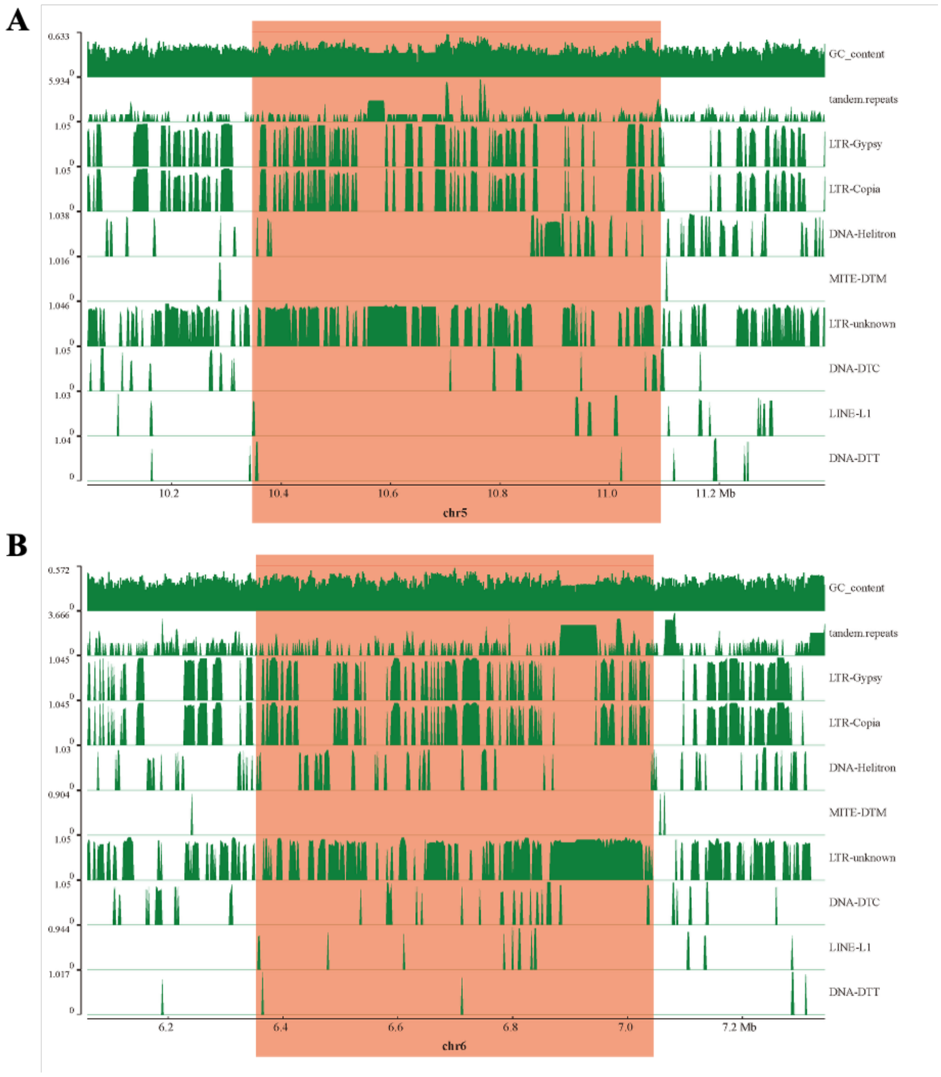


**Supplementary Figure S15 The distribution of repetitive sequences in the centromeric region of chr5 and chr6 chromosomes in *E. cheiranthoides* (Zhai *et al*., 2024). (A)** GC content and TE distribution of the centromere region of chr5 chromosome. **(B)** GC content and TE distribution of the centromere region of chr6 chromosome.

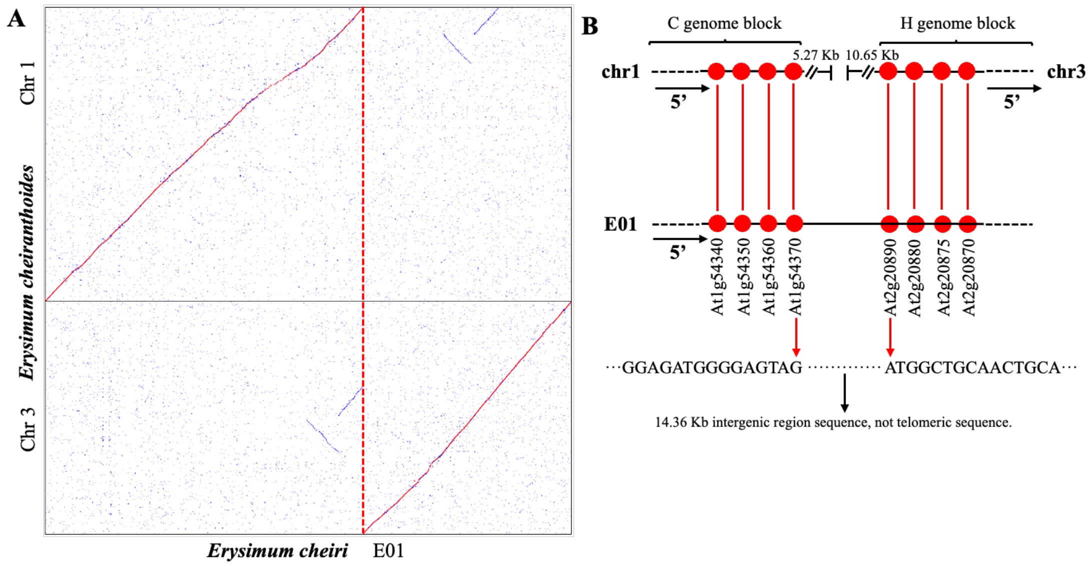


**Supplementary Figure S16 The end-to-end chromosome translocation (EET) model of the chr1 connected with chr3 of *E. cheiranthoides* (Zhai *et al*., 2024) forms E01 of *E. cheiri*.** **(A)** Collinearity between chr1, chr3 and E01 chromosomes. **(B)** The details of the chr1 and chr3 junction site on chromosome E01 in *E. cheiri*. Red dots and lines indicate homologous genes in both species, whereas gray dots indicate that no homologous genes have been identified. 5.27 Kb is the telomeric sequence of chr1, and 10.65 Kb is the telomeric sequence of chr3.

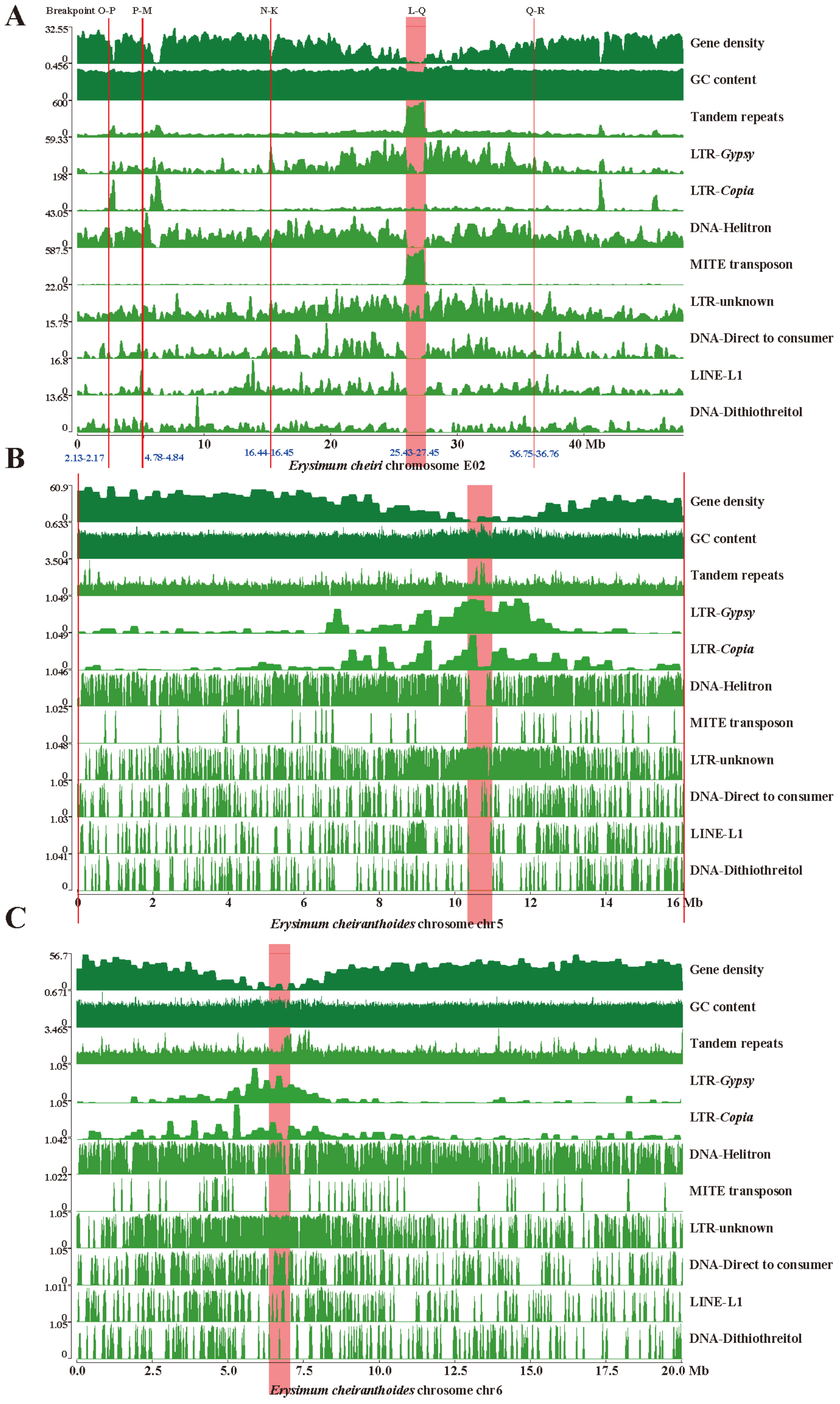


**Supplementary Figure S17 The distribution of gene density, GC content, and repetitive sequences on chromosomes E02 of *E. cheiri* (A), and chr5 (B) and chr6 (C) of *E. cheiranthoides*.** Window size, 200 kb. The light red and red vertical bars represent the centromeres and fusion breakpoint, respectively. The blue numbers indicate the start and end positions of the breakpoints.

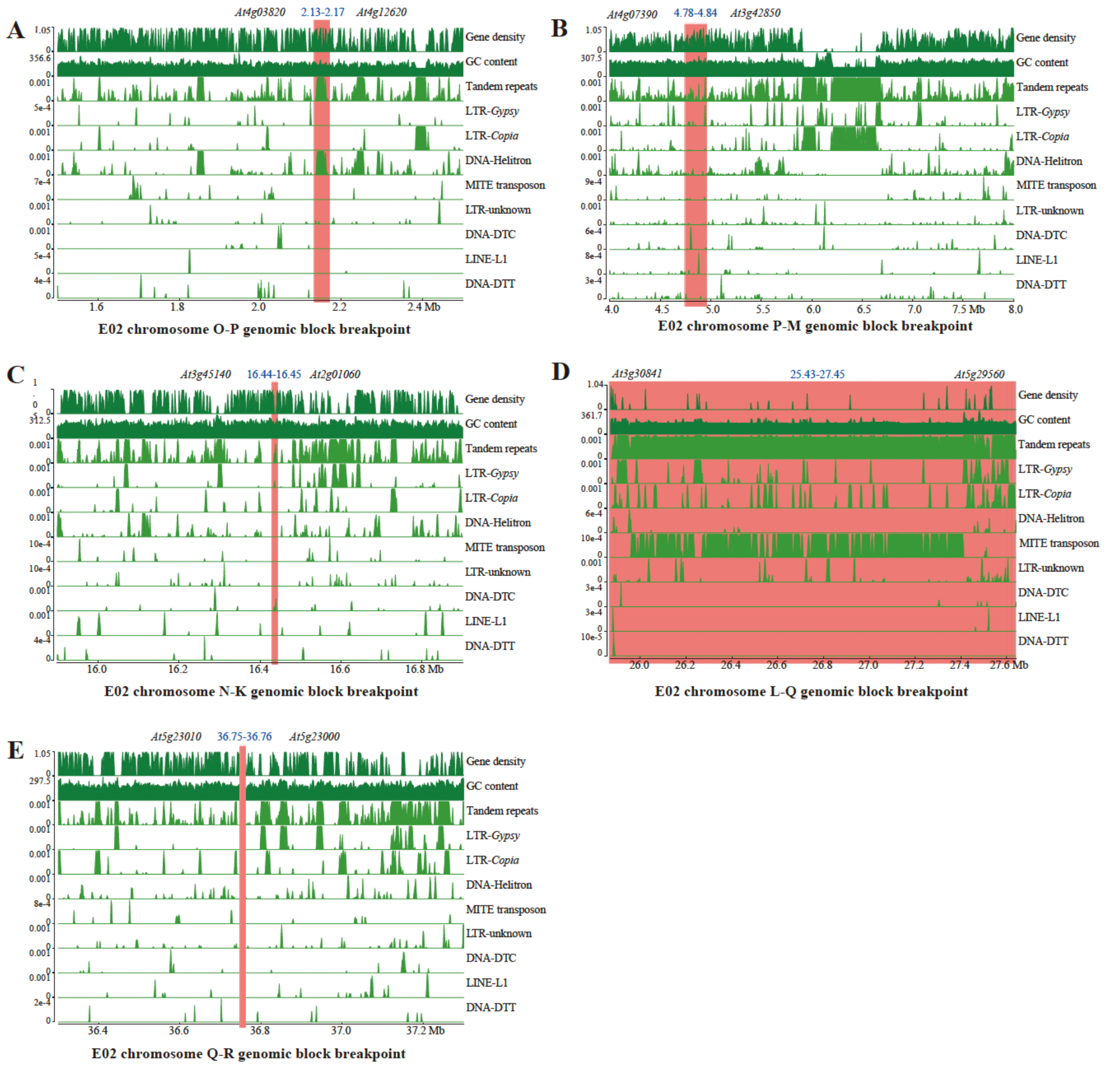


**Supplementary Figure S18 The distribution of gene density, GC content, and repetitive sequences of five breakpoint on chromosomes E02 of *E. cheiri*. (A)** O-P genomic block breakpoint. **(B)** P-M genomic block breakpoint. **(C)** N-K genomic block breakpoint. **(D)** L-Q genomic block breakpoint. **(E)** Q-R genomic block breakpoint. Window size, 1 kb. The light red vertical bars represent the fusion breakpoints, and the blue values and *Arabidopsis thaliana* genes define the breakpoint start and end positions.

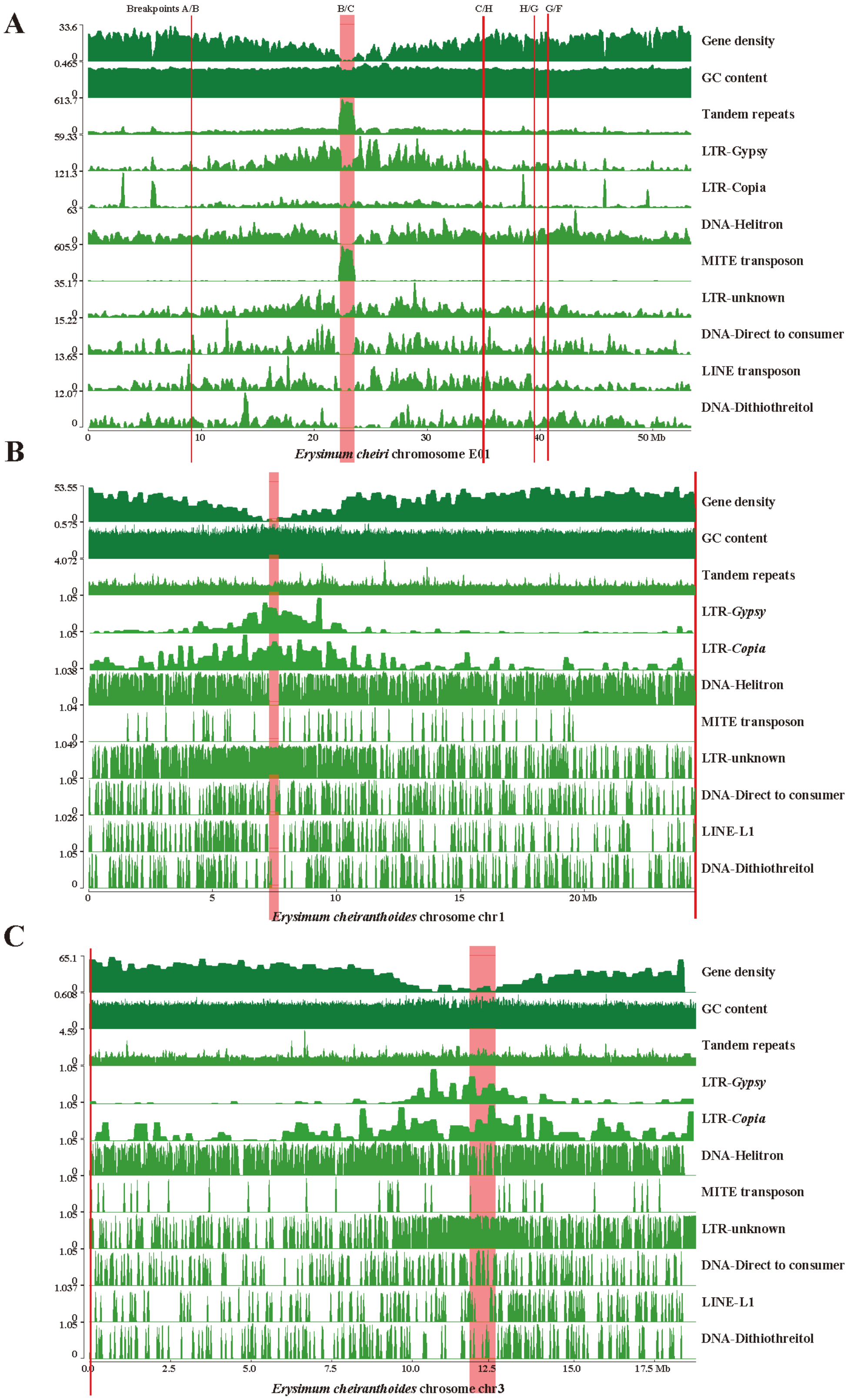


**Supplementary Figure S19 The distribution of gene density, GC content, and repetitive sequences on chromosomes E01 of *E. cheiri* (A), and chr1 (B) and chr3 (C) of *E. cheiranthoides*.** Window size, 200 kb. The light red and red vertical bars represent the centromeres and fusion breakpoint, respectively.

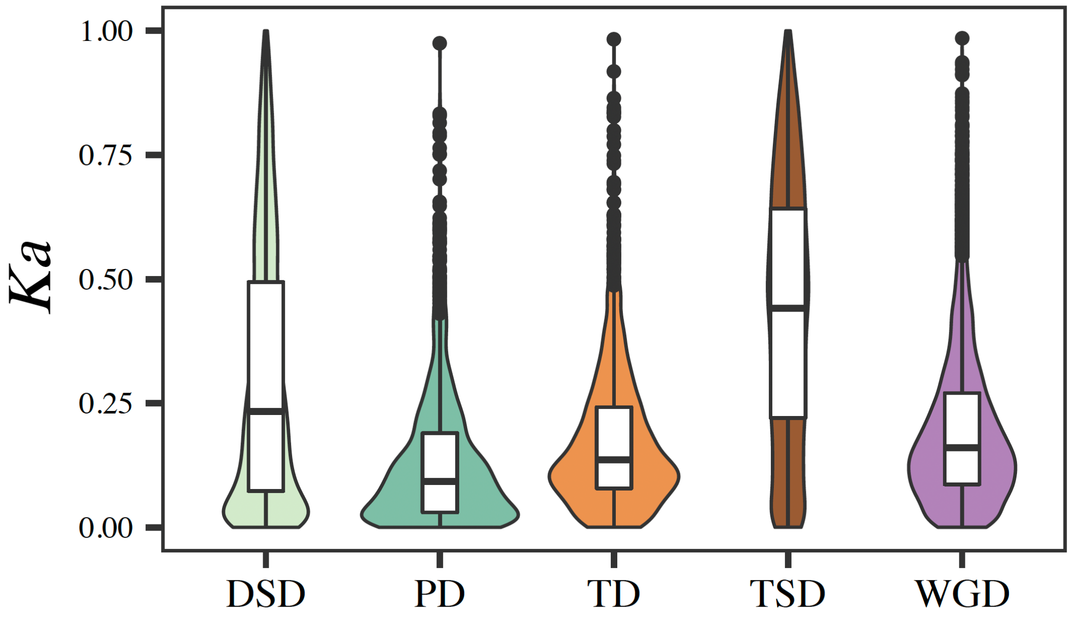


**Supplementary Figure S20 The frequency distributions of non-substitutions (*Ka*) for five types of gene duplication.** DSD: dispersed duplicated genes, PD: proximal duplicated genes, TD: tandem duplicated genes, TSD: transposed duplicated genes, WGD: whole-genome duplicated genes. In the violin plots, the center black lines represent the medians, the box limits correspond to the 25th and 75th percentiles, the whiskers extend to 1.5 times the interquartile ranges, the shapes indicate the data distributions.

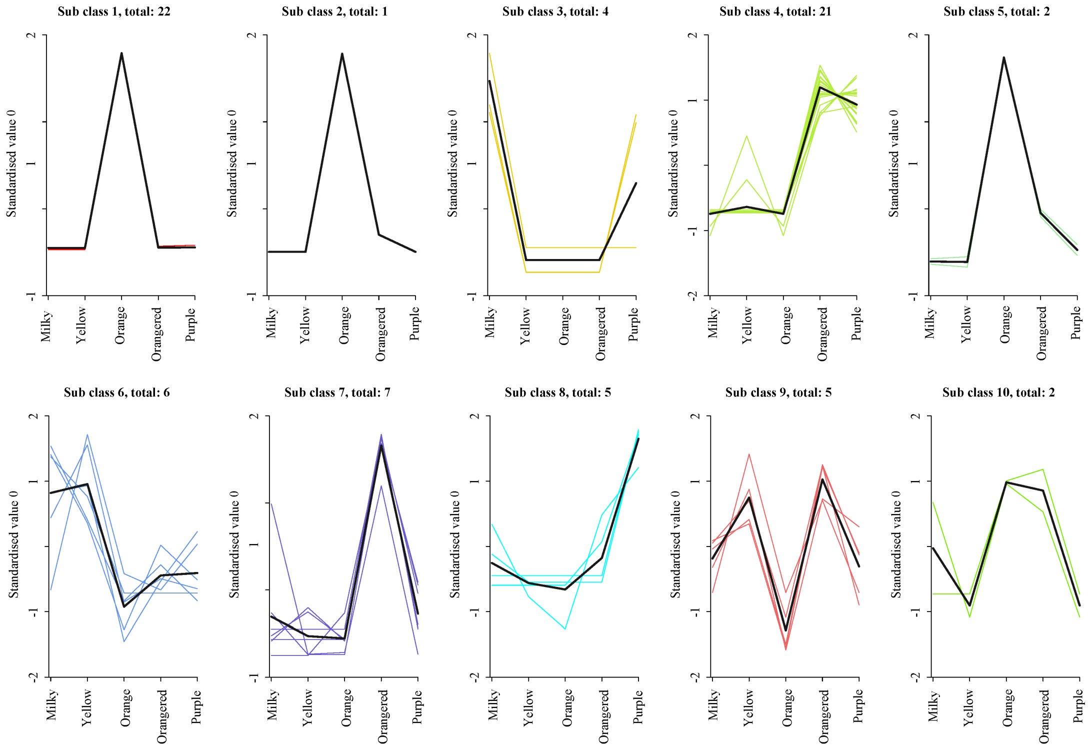


**Supplementary Figure S21 The *K-means* cluster analysis diagram for anthocyanins.**

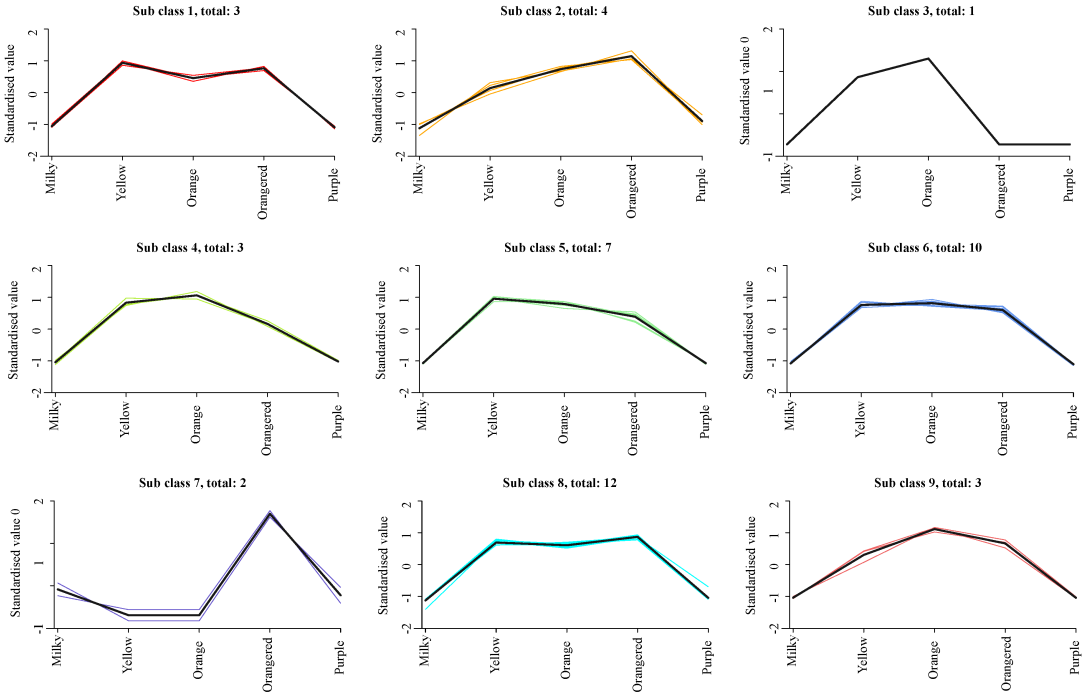


**Supplementary Figure S22 The *K-means* cluster analysis diagram for carotenoids.**

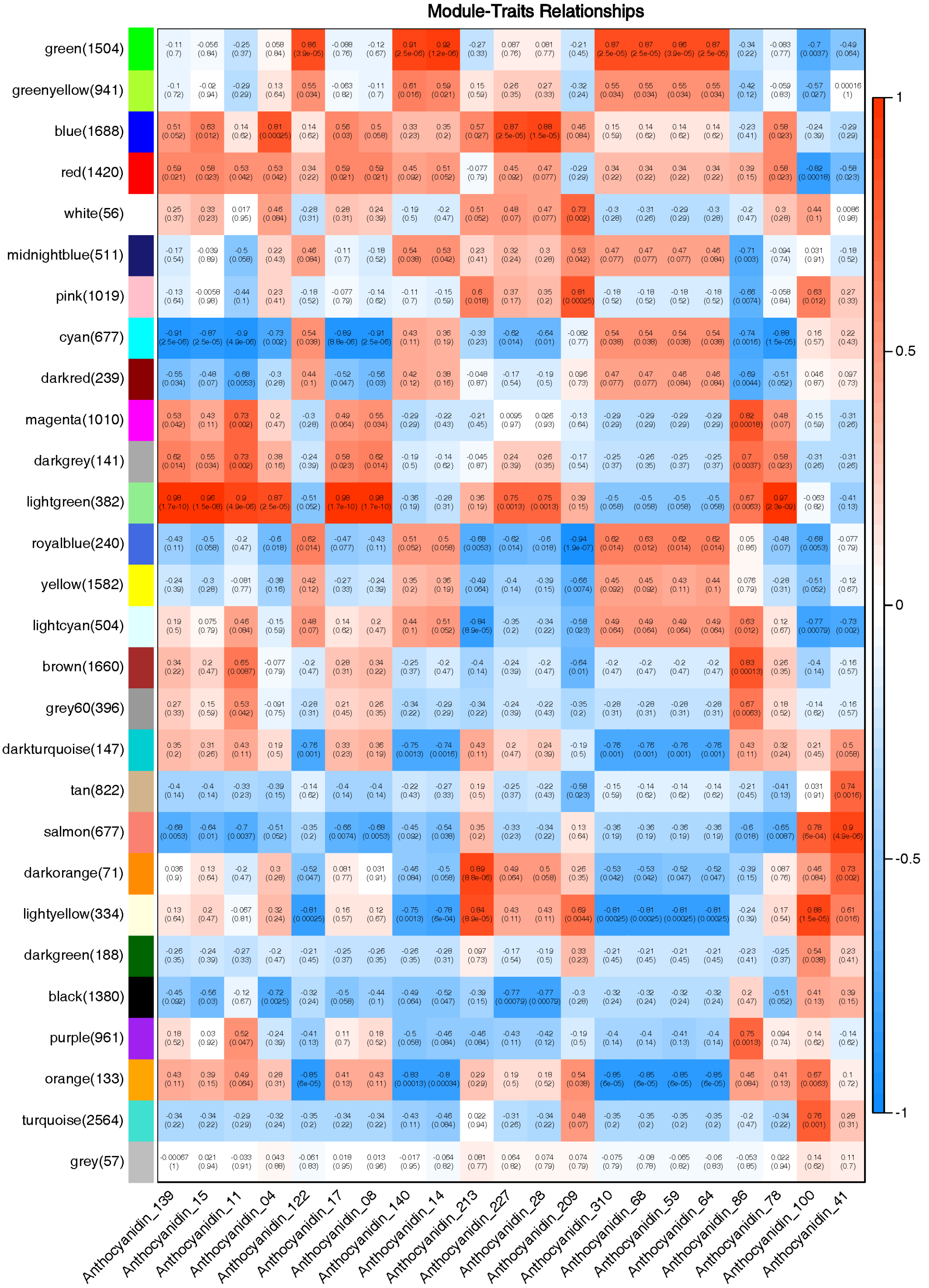


**Supplementary Figure S23 Weighted Correlation Network Analysis (WGCNA) between gene expression and anthocyanin content of *E. cheiri* flower petals.**

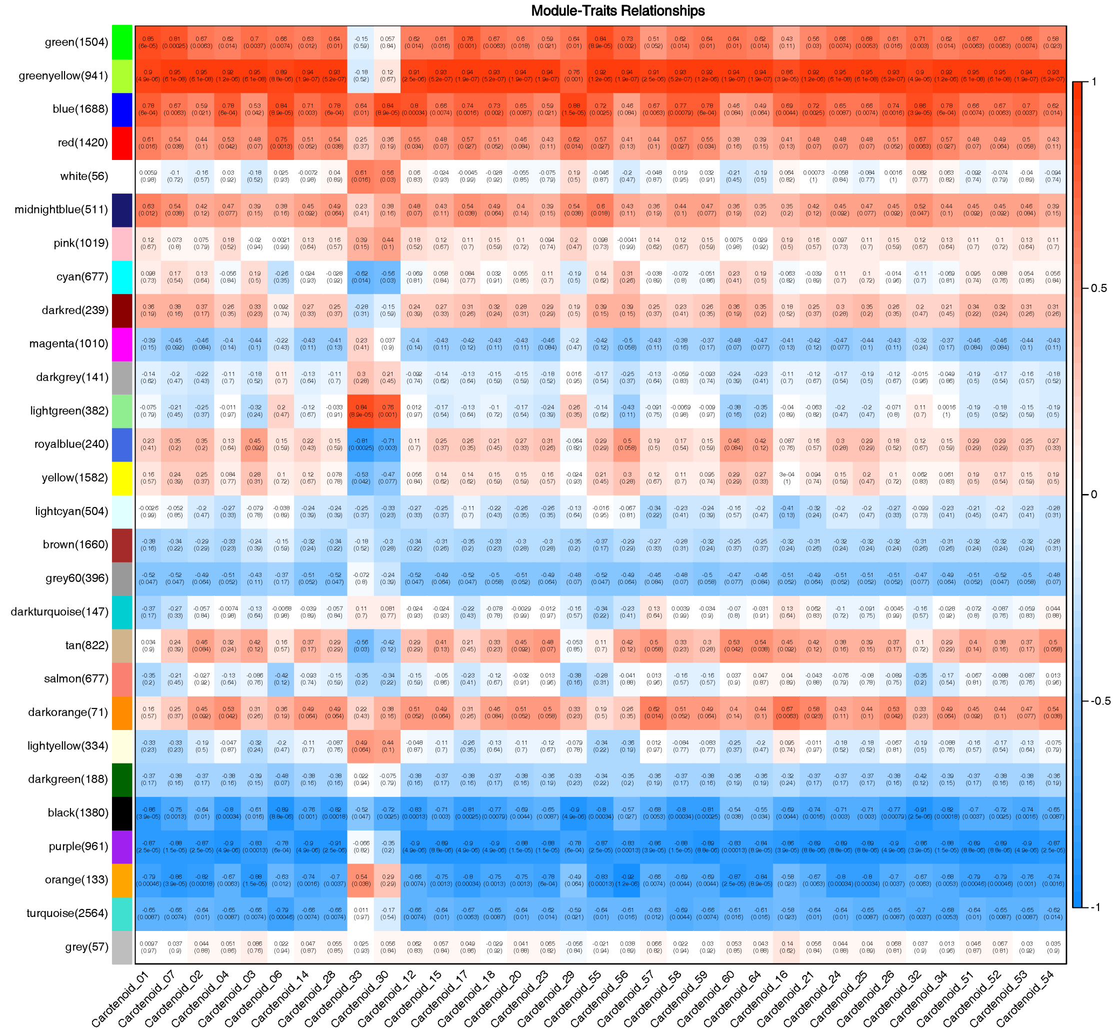


**Supplementary Figure S24 WGCNA analysis between gene expression and carotenoid content of *E. cheiri* flower petals.**
